# Prediction and Rationalization of Different Photochemical Behaviors of *mer*- and *fac*-Isomers of [Ru(pyridyltriazole)_3_]^2+^

**DOI:** 10.1021/acs.inorgchem.4c03154

**Published:** 2024-09-05

**Authors:** Paul A. Scattergood, Paul I. P. Elliott

**Affiliations:** Department of Physical and Life Sciences & Centre for Functional Materials, University of Huddersfield, Queensgate, Huddersfield HD1 3DH, U.K.

## Abstract

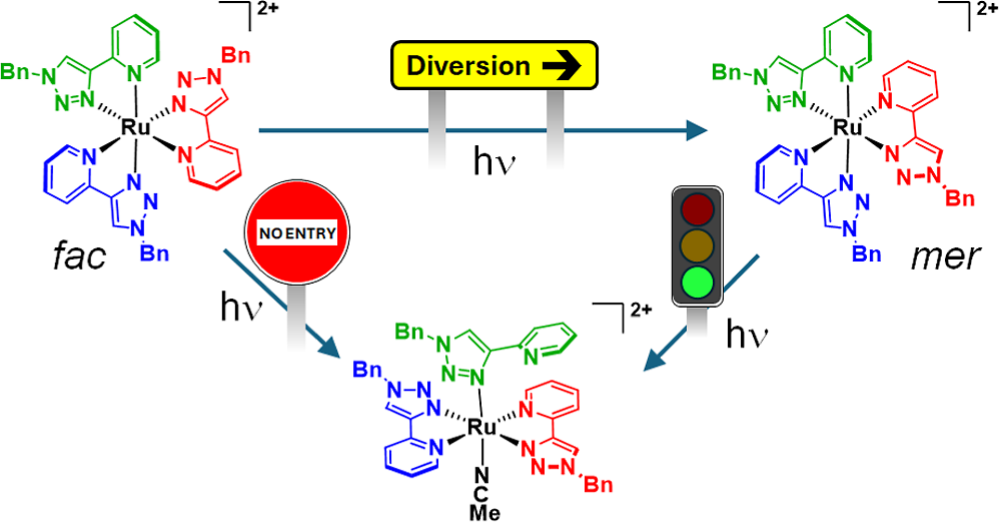

Facial and meridional isomerism of metal complexes is known to result in fundamental differences in photophysical properties. One may also envisage differences in their photochemical reactivity and therefore predict different outcomes of their light-triggered transformations. The *fac*- and *mer*-isomers of the complex [Ru(pytz)_3_]^2+^ (*fac*-**1** & *mer*-**1**, pytz = 1-benzyl-4-(pyrid-2-yl)-1,2,3-triazole) were separated and isolated. *mer*-**1** undergoes a predicted pytz photodechelation process in acetonitrile to yield *trans*-[Ru(κ^2^-pytz)_2_(κ^1^-pytz)(NCMe)]^2+^ (**2**) whereas unfavorable interligand steric interactions are predicted to, and indeed do prevent comparable photoreactivity for *fac*-**1**. Reversible photoisomerization of *fac*-**1** and *mer*-**1** is also observed, however. The differences in photochemical reactivity of the two isomers can be rationalized based on structural programming of the preferential accessibility of particular ^3^MC excited states due to differences in their interligand steric interactions. Here we present an initial predictive thought experiment, subsequent experimental verification, and computational rationalization of the differences in photochemical reactivity of these two isomeric complexes.

## Abstract 2

Understanding of geometry and intermolecular steric factors at play in particular ^3^MC states enables informed prediction of differing photochemical reactivity and outcomes for isomers of Ru(II) complexes.

## Introduction

Kinetically inert d^6^ metal complexes have attracted enormous interest in the literature over recent decades due to their photophysical properties which have seen their application in light-emitting device technologies,^1^ dye-sensitized solar cells,^2^ luminescence imaging microscopy,^3,4^ photodynamic therapy,^5,6^ and photocatalysis.^7^ Key to successful employment in these applications are the relatively long-lived triplet metal-to-ligand charge transfer (^3^MLCT) excited states that are populated following photoexcitation.^8^ However, Ru(II) complexes are also of interest in photoactivated chemotherapy,^9−11^ in which the triplet metal centered (^3^MC) states (that may act to rapidly deactivate ^3^MLCT states) are harnessed to promote ligand dissociation photochemistry^12−19^ resulting in the formation of cytotoxic fragments. For example, the sterically encumbered complex [Ru(bpy)_2_(6,6′-dmbpy)]^2+^ (bpy = 2,2′-bipyridyl; 6,6′-dmbpy = 6,6′-dimethyl-2,2′-bipyridyl) undergoes rapid photolysis in donor solvents to yield *cis*-[Ru(bpy)_2_(solvent)_2_]^2+^ and free 6,6′-dmbpy, which when initiated in cancer cells induces potent phototoxicity.^20^

While the majority of examples involve formation of photoproducts with *cis* stereochemistry, there are examples in which photoproducts with *trans* stereochemistry are also observed. For example, the complex [Ru(deeb)(bpz)_2_](Br)_2_ (deeb = 2,2′-bipyridyl-4,4′-dicarboxylate, bpz = 2,2′-bipyrazinyl) undergoes photolysis in acetone to yield both *cis*- and *trans*-[Ru(deeb)(bpz)(Br)_2_] via the intermediate [Ru(deeb)(κ^2^-bpz)(κ^1^-bpz)(Br)].^21^ We have previously reported the photochemistry of the complex [Ru(bpy)(btz)_2_]^2+^ (btz = 1,1′-dibenzyl-4,4′-bi-1,2,3-triazolyl).^22,23^ In acetonitrile solutions the complex photochemically liberates one btz ligand, undergoing a rearrangement of the remaining bidentate ligands which become coplanar to yield the photoproduct *trans*-[Ru(bpy)(btz)(NCMe)_2_]^2+^. Significantly, this proceeds with the observation of the ligand-loss intermediate *trans*-[Ru(bpy)(κ^2^-btz)(κ^1^-btz)(NCMe)]^2+^ in a rapid and efficient first step which proceeds with a photochemical quantum yield of 0.34.^24^ Crystallographically characterized complexes of the form *trans*-[Ru(bpy)_2_(L)_2_]^2+^ reveal severe distortions away from coplanarity of the two bpy ligands due to the steric clash between the ligands imparted by the pyridine 6-position hydrogen atoms.^25,26^ However, such a steric clash is absent for these btz-containing complexes enabling the observed and remarkably facile coplanarisation process.

**Figure 1:**
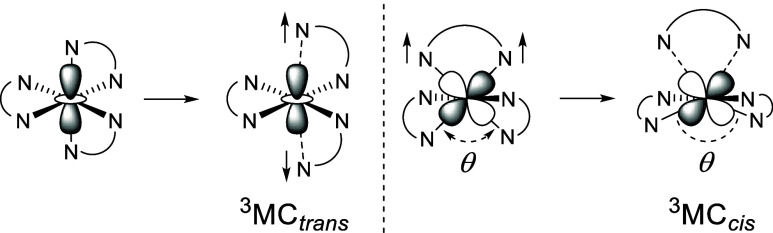
Geometric distortions in *tris*-chelate complexes arising through population of d_*z*^2^_-like and d_*x*^2^–*y*^2^_-like dσ* orbitals and population of ^3^MC_trans_ and ^3^MC_cis_ states respectively.

Insights into the electronic structure and geometry of ^3^MC states in [Ru(N^N)_3_]^2+^-type complexes have been facilitated in the past two decades through computational studies. Early results revealed ^3^MC states exhibiting an axial elongation of two *trans* Ru–N bonds (^3^MC_trans_)^27,28^ involving accommodation of the excited electron in a d_*z*^2^_-like dσ* orbital (Figure 1). More recent results have revealed the ^3^MC region of the triplet excited state potential energy surface of these complexes to be an expansive basin exhibiting numerous minima.^29^ Different possible ^3^MC states may then favor different outcomes with respect to ground state recovery or photochemical reactivity. Density functional theory (DFT) studies of [Ru(bpy)(btz)_2_]^2+^ revealed the existence of a previously unrecognized class of ^3^MC state characterized by population of a d_x^2^–y^2^_-like dσ* orbital, and featuring elongation of both Ru–N bonds to the same btz ligand (^3^MC_cis_) with a concomitant widening of the angle between the planes of the other two ligands toward coplanarisation (Figure 1).^30^ The observed photoproduct stereochemistry therefore stems from a combination of geometric factors in the ^3^MC_cis_ state and interligand steric interactions (or more to the point, the lack thereof). In the case of [Ru(deeb)(bpz)_2_](Br)_2_ the detected intermediate is suspected, but not fully confirmed, to have *cis*-stereochemistry with the *trans* photoproduct forming through subsequent isomerization of the *cis* product.^21^

We have also shown that a ^3^MC_cis_ state exists and is accessible for [Ru(bpy)_3_]^2+^.^31,32^ Indeed, the ^3^MC_cis_ state was found to be only 2 kcal mol^–1^ higher in energy than the ^3^MC_trans_ state for [Ru(bpy)_3_]^2+^ with a comparable barrier to population from the ^3^MLCT state. Further, the singlet–triplet minimum energy crossing point (^1,3^MECP) for the ^3^MC_cis_ state was found to be 3 kcal mol^–1^ lower in energy that for the ^3^MC_trans_ state. Thus, the ^3^MC_cis_ state for [Ru(bpy)_3_]^2+^ will inevitably play a previously unknown and key role in the experimentally observed temperature dependent ^3^MLCT state deactivation to the electronic ground state. This has subsequently been corroborated by Gonzalez and Rau in reassessing the thermal dependence of ^3^MLCT state deactivation in light of our work.^33^ Further, using [Ru(bpy)_3_]^2+^ as a model system for ligand release photochemistry, it was shown to be feasible for the ^3^MC_cis_ state to contribute to the formation of *cis* as well as *transbis*-solvent photoproduct formation pathways.^32^ In more recent studies we were able to show that Ru(bpy)_2_-containing complexes in which a ^3^MC_cis_ state can be located on the T_1_ potential energy surface show enhanced photochemical quantum yields for the formation of *cis*-[Ru(bpy)_2_(NCMe)_2_]^2+^ in acetonitrile.^34^ On the other hand, while incorporation of a single sterically straining ligand methyl substituent promotes significant stabilization of ^3^MC_trans_ states and enhanced ^3^MLCT state deactivation, no ^3^MC_cis_ state can be located on the T_1_ surface and complexes typically fail to exhibit significantly enhanced photochemical ligand release. We concluded, therefore, that ^3^MC_cis_ states in *tris*-chelate complexes are far more prone to photochemical reactivity, even for the formation of *cisbis*-solvento products, whereas ^3^MC_trans_ states favor ground state recovery and may in fact act to quench photochemistry.

**Scheme 1:**
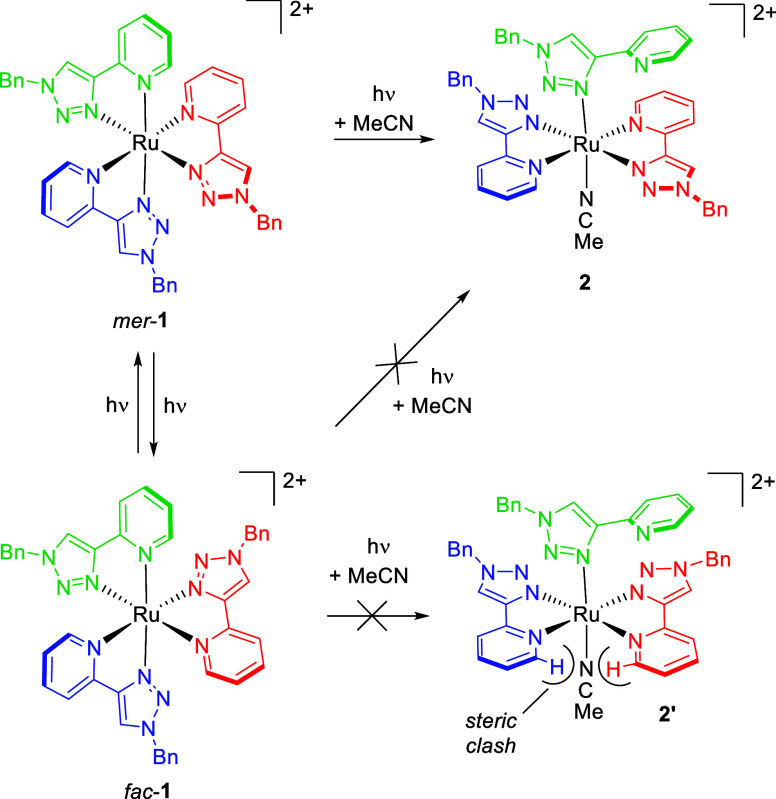
Predicted Outcomes of Photochemistry for *mer*- and *fac*-[Ru(pytz)_3_]^2+^ in Acetonitrile

These insights from our previous work enable experimentally verifiable predictions of photochemical reactivity to be made. The homoleptic complex [Ru(pytz)_3_]^2+^ (**1**, pytz = 1-benzyl-4-(pyrid-2-yl)-1,2,3-triazole) represents one such system.^35−37^ Homoleptic complexes of asymmetric ligands such as pytz lead to the formation of mixtures of facial and meridional isomers,^38,39^ which in the case of [Ru(pytz)_3_]^2+^ have been shown by Crowley and others to be conveniently separated by chromatographic methods.^36,40,41^ Due to the lack of symmetry in the complex the three ligands in *mer*-[Ru(pytz)_3_]^2+^ (*mer*-**1**) are rendered inequivalent (and are color-coded green, blue and red in Scheme 1). Consequently, the complex will potentially exhibit three unique ^3^MC_trans_ states and three unique ^3^MC_cis_ states. It can be seen that if the green pytz ligand is repelled in a ^3^MC_cis_ state (*mer*-^3^MC_cis_,_green_), the angle between the blue and red ligands will widen and the two ligands will approach coplanarity (the same is true if the blue ligand is repelled in a *mer*-^3^MC_cis,blue_ state). With no steric clash between ligands, *mer*-**1** would be expected to exhibit favorable formation of the *trans* photoproduct *trans*-[Ru(κ^2^-pytz)(κ^1^-pytz)(NCMe)]^2+^ (**2**).

If, on the other hand, the red pytz ligand of *mer*-**1** is repelled (*mer*-^3^MC_cis_,_red_) the blue and green pyridine rings will be brought toward a *cis* arrangement resulting in a steric clash, thus inhibiting access to a *trans* photoproduct. However, rotation of the red ligand by 180° as a result of population of this state could result in *mer*/*fac* isomerization. When one similarly considers *fac*-**1** the three pytz ligands are equivalent and the formation of a *trans* photoproduct (**2′**) will always be disfavored.

While it is feasible that *fac*-**1** may undergo photochemical pytz release to form *cis*-[Ru(pytz)_2_(NCMe)_2_]^2+^ or photoisomerisation to yield the *mer*-isomer (as is well-known for cyclometalated iridium(III) complexes^42−44^), it will not be able to readily form *trans*-[Ru(pytz)_2_(NCMe)_2_]^2+^ (**2**′) due to unfavorable steric interactions. However, *mer*-**1** can be confidently predicted to be far more photochemically reactive than its *fac* isomer and should readily and selectively form **2**. We present here the experimental exploration and verification of these predictions as well as a detailed computational study of the triplet excited state reactivity of this *fac*/*mer* system.

## Results & Discussion

**Figure 2:**
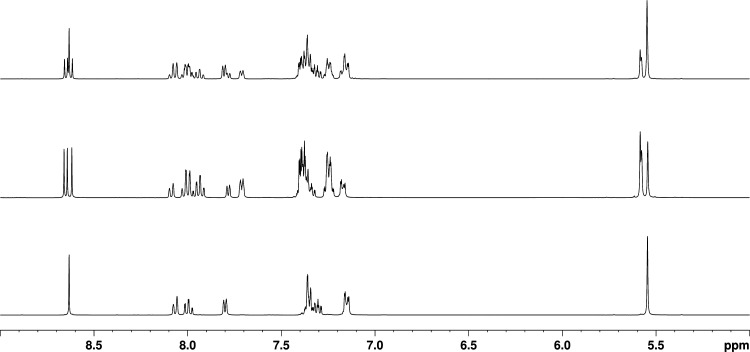
^1^H NMR spectrum of *fac*/*mer*-[Ru(pytz)_3_]^2+^ (**1**, top) in *d*_3_-acetonitrile and those of the separated isomers *mer*-[Ru(pytz)_3_]^2+^ (*mer*-**1**, center) and *fac*-[Ru(pytz)_3_]^2+^ (*fac*-**1**, bottom) in the same solvent.

[Ru(pytz)_3_]^2+^ was prepared as a mixture of *mer*- and *fac*-isomers as the hexafluorophosphate salt by refluxing the precursor [Ru(η^6^-cymene)(pytz)(Cl)][PF_6_] with two equivalents of pytz in ethanol/water followed by counteranion metathesis. Separation of the *mer*- and *fac*-isomers was then carried out by thin layer chromatography using dichloromethane as an initial eluent followed by dichloromethane/acetone.^40^ The ^1^H NMR spectrum of *fac*-**1** exhibits one singlet resonance for the three equivalent triazole ring protons at δ 8.63 (Figure 2). Due to the inequivalence of the three pytz ligands, the ^1^H NMR spectrum of *mer*-**1** is more complicated and features three distinct singlet resonances at δ 8.62, 8.64 and 8.66 for the triazole ring protons of each ligand.

Electrochemical data from cyclic voltammetry (Figure S5 and Table 1) reveal reversible oxidation processes for *fac*-**1** and *mer*-**1** at +0.96 and +0.94 V (versus Fc^+^/Fc) respectively, assigned to a Ru(II)/Ru(III) oxidation process. Both complexes show two irreversible reduction process at approximately −2.2 and −2.4 V (versus Fc^+^/Fc) assigned to reduction of the pytz ligands. These ligand reductions are cathodically shifted by approximately 0.5 V compared to the bpy-centered reductions for [Ru(bpy)_3_]^2+^ illustrating the significantly higher energy of the pytz-centered LUMO in these complexes. These data agree with those previously reported for the mixture of isomers and for the separated isomers.^36^ DFT calculations on the optimized ground state geometries (see Table 2 for structural parameters and Supporting Information) of *fac*-**1** and *mer*-**1** show that the HOMO in both cases is primarily composed of the ruthenium d_*z*^2^_ orbital while the LUMO has ligand π* character and is mostly localized on the pyridine rings (Figure S6). In both cases HOMO-1 and HOMO-2 also have d-orbital character with HOMO-3 to HOMO-5 having ligand π-character. Above the LUMO, LUMO + 1 to LUMO + 5 have ligand π* character which is primarily localized on the pyridine rings of the ligands while LUMO + 6 to LUMO + 8 have triazole ring π* character. For both complexes, the LUMO + 9 and LUMO + 10 orbitals are dσ* Ru–N antibonding in nature.

**Figure 3:**
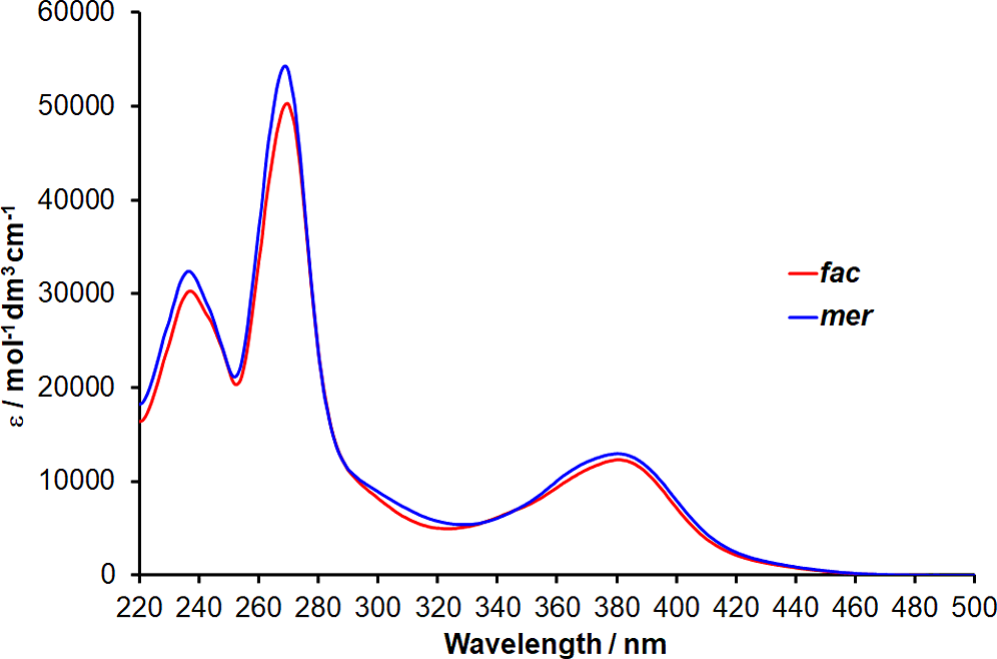
UV–visible absorption spectra of *fac*-**1** and *mer*-**1** in acetonitrile.

The separated isomers exhibit near identical UV–visible absorption spectra in acetonitrile (Figure 3) in agreement with previous studies.^36,40,41^ Spectra feature a prominent band at ∼380 nm assigned to ^1^MLCT transitions with intense bands in the UV at ∼270 and 240 nm assigned to ^1^LC transitions for the pytz ligands. Predicted absorption spectra from TDDFT calculations are in fairly good agreement with experimental spectra (Figure S7) and confirm assignment of these bands (Table S1). The prominent band between 360 and 420 nm is calculated to arise primarily through ^1^MLCT transitions with localization of the excited electron on the pyridine rings of the pytz ligands (LUMO to LUMO + 5). Lower intensity absorptions centered at 300 nm observable in the experimental spectra are also calculated to have ^1^MLCT character but with acceptor orbitals localized on the triazole rings (LUMO + 6 to LUMO + 8). The ^1^MLCT absorption bands are blue-shifted by about 70 nm relative to the corresponding band for [Ru(bpy)_3_]^2+^, again reflecting the destabilization of the pytz-centered LUMO relative to the bpy-centered LUMO, and a significantly increased HOMO–LUMO energy gap.

Neither isomer was found to exhibit detectable luminescence either in room temperature acetonitrile solutions or in a frozen solvent glass at 77 K. This indicates that the ^3^MLCT states of the complexes are rapidly deactivated, presumably by population of ^3^MC states, even at low temperature. Optimized T_1_ states were determined starting at the optimized ground state geometries and are found to reside 2.5 eV above the ground states (Table 2). They are thus heavily destabilized relative to the ^3^MLCT state of the archetypal ruthenium(II) complex [Ru(bpy)_3_]^2+^ (2.03 eV calculated using identical parameters^34^) which would indeed be expected to facilitate rapid ^3^MC state-mediated depopulation and result in a significantly enhanced rate of nonradiative deactivation. A T_1_ state of ^3^MLCT state character is found for *fac*-**1** (Table 2 and Supporting Information) exhibiting a lower energy singly occupied natural orbital (SONO) of predominantly metal d-orbital character and a higher energy SONO of ligand π* character localized largely on the pyridine ring of one of the pytz ligands (Figure S8). For the T_1_ state of *mer*-**1** a spin density of 0.97 is obtained consistent with ^3^MLCT character. Both SONOs have ligand as well as metal character (the green pytz ligand depicted in Table 2) indicating possible admixing of ^3^LC and/or ^3^MC character in the ^3^MLCT state.

**Table 1:** Summarised UV–Visible Absorption Spectral Data (in Acetonitrile) and Electrochemical Data (Versus Fc/Fc^+^ = 0.0 V, Recorded at 100 mV s^–1^ in Acetonitrile Containing ^*n*^NBu_4_PF_6_ Electrolyte) for *fac*-**1** and *mer*-**1**

complex	λ^abs^/nm (ε/dm^3^ mol^–1^ cm^–1^)	*E*_ox_/V (*E*_a_ – *E*_p_/mV)	*E*_red_/V
*fac*-**1**	238 (30,150)	+0.96 (76)	–2.20 (irr)
	270 (50,170)		–2.40 (irr)
	382 (12,280)		
*mer*-**1**	237 (32,380)	+0.94 (80)	–2.22 (irr)
	268 (54,080)		–2.45 (irr)
	379 (12,940)		

**Table 2:**
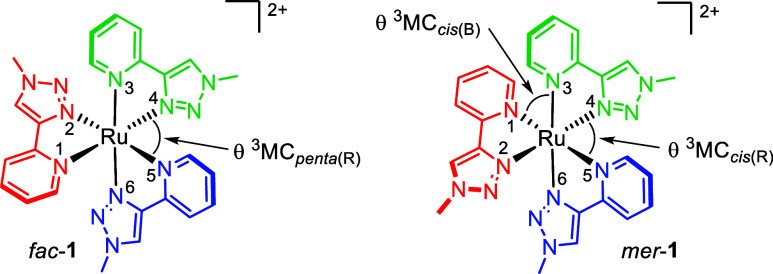
Selected Structural Parameters, Energies and Ru Atom Spin Densities for the ^1^GS, ^3^MLCT and ^3^MC Electronic States of *fac*-**1** and *mer*-**1** Obtained from DFT Calculations^a^

*fac*-[Ru(pytz)_3_]^2+^				
	^1^GS	^3^MLCT	^3^MC_trans_	^3^MC_penta(R)_
energy	0.00	2.51	2.13	2.29
Ru spin density	0.00	0.91	1.91	1.77
Ru–N(1)	2.10	2.03	2.11	**2.21**
Ru–N(2)	2.05	2.04	2.06	**3.89**
Ru–N(3)	2.10	2.11	**2.54**	2.10
Ru–N(4)	2.04	2.08	2.09	2.05
Ru–N(5)	2.11	2.11	2.14	2.21
Ru–N(6)	2.04	2.04	**2.49**	2.05
N–Ru–N θ				131.7

*mer*-[Ru(pytz)_3_]^2+^							
	^1^GS	^3^MLCT	^3^MC_trans(R/G)_	^3^MC_trans(R/B)_	^3^MC_trans(B/G)_	^3^MC_cis(R)_	^3^MC_cis(B)_
energy	0.00	2.50	2.11	2.03	2.07	2.10	2.15
Ru spin density	0.00	0.97	1.91	1.89	1.91	1.77	1.79
Ru–N(1)	2.10	2.07	2.14	**2.51**	2.11	**2.58**	2.12
Ru–N(2)	2.04	2.05	**2.54**	2.11	2.07	**2.29**	2.05
Ru–N(3)	2.10	2.02	2.12	2.10	**2.54**	2.10	2.27
Ru–N(4)	2.05	2.06	**2.45**	2.05	2.10	2.08	2.07
Ru–N(5)	2.10	2.16	2.11	**2.51**	2.13	2.24	**2.23**
Ru–N(6)	2.04	2.11	2.07	2.08	**2.49**	2.06	**2.63**
N–Ru–N θ						132.2	119.4

a Energies are quoted in eV relative to the electronic ground state of the parent isomer in each case. Structures of *fac*-**1** and *mer*-**1** are provided to illustrate the atom numbering system. The subscript letters R, B and G refer to the ligands predominantly participating in bond elongations in ^3^MC states based on their colors [red (R), green (G) and blue (B)] within the depicted structures. Pertinent bond length (Å) elongations are shown in bold, while angles (θ) are given in degrees.

**Scheme 2:**
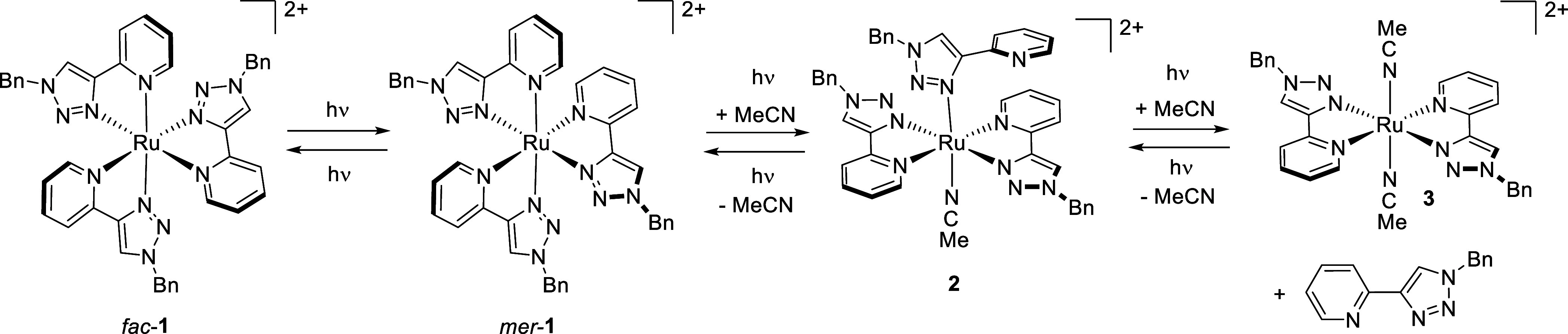
Proposed Photochemical Reactivity of *fac*-**1** and *mer*-**1** in Acetonitrile

**Figure 4:**
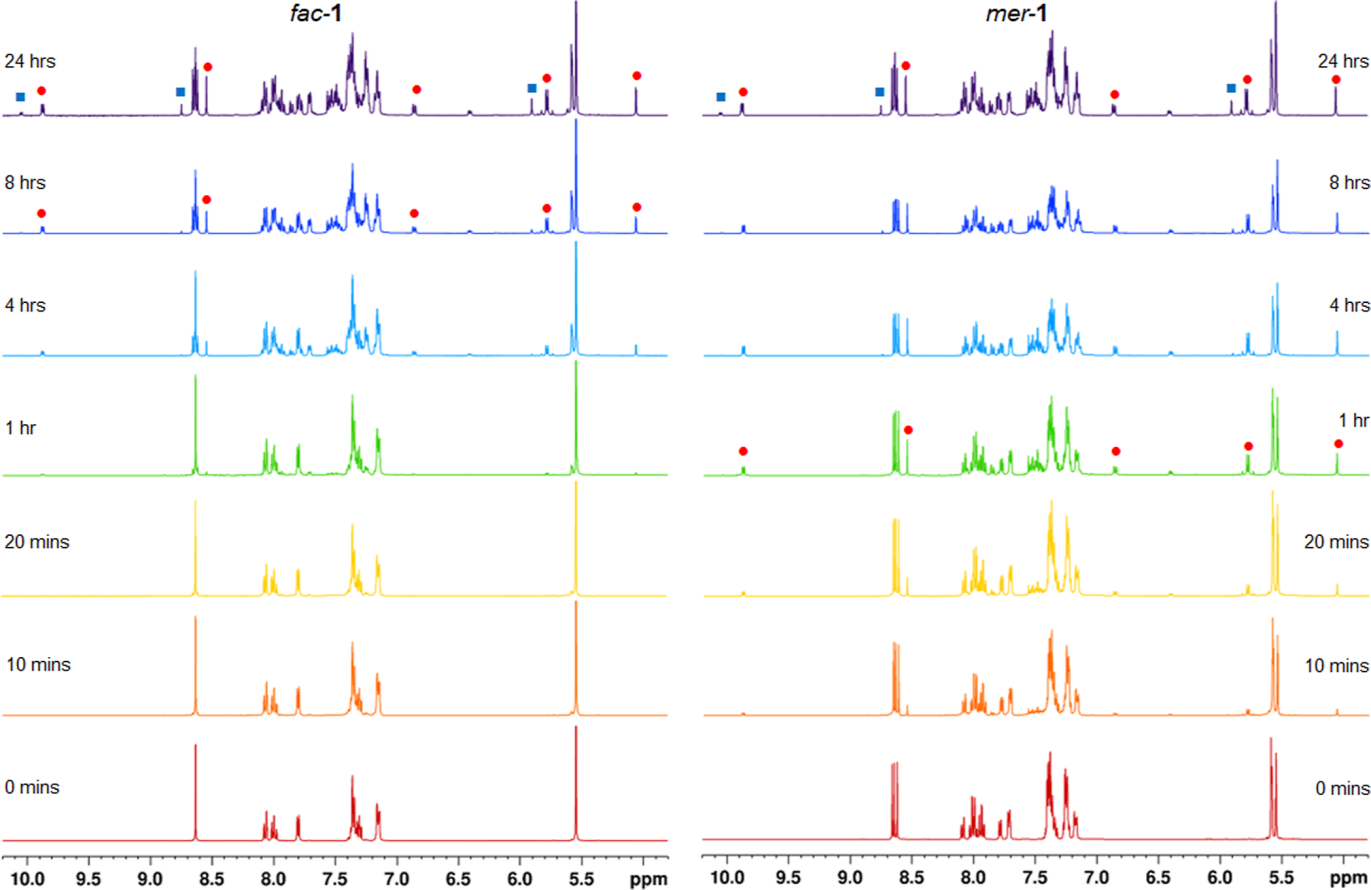
^1^H NMR spectra recorded during the photolysis of *fac*-**1** and *mer*-**1** in *d*_3_-acetonitrile (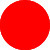 = *trans*-[Ru(κ^2^-pytz)_2_(κ^1^-pytz)(NCCD_3_)]^2+^, 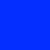 = *trans*-[Ru(pytz)_2_(NCCD_3_)_2_]^2+^).

The photochemistry of *fac*-**1** and *mer*-**1** in d_3_-acetonitrile using irradiation from a 23 W domestic lamp (Figure S9) was monitored by ^1^H NMR spectroscopy. After 20 min of irradiation the ^1^H NMR spectrum of a sample of *mer*-**1** exhibits multiple additional resonances demonstrating photochemical conversion. These include a new and significantly deshielded doublet resonance at δ 9.88 and a singlet at δ 8.55 (Figure 4). Based on the similarity to features observed in ^1^H NMR spectra of *trans*-[Ru(bpy)(κ^2^-btz)(κ^1^-btz)(NCMe)]^2+^^22,23^ these resonances are assigned to the pyridine-H6 and triazole ring protons respectively of the two κ^2^-pytz ligands of the ligand-loss intermediate *trans*-[Ru(κ^2^-pytz)_2_(κ^1^-pytz)(NCMe)]^2+^ (**2**, Scheme 2). These resonances are accompanied by the observation of a roofed pair of doublets at δ 5.80 and 5.77 (*J*_HH_ = 14.8 Hz) assigned to the benzylic methylene protons of the κ^2^-pytz ligands of **2**, again, highly reminiscent of the analogous resonances for the corresponding protons of the κ^2^-btz ligand in *trans*-[Ru(bpy)(κ^2^-btz)(κ^1^-btz)(NCMe)]^2+^. The benzylic methylene protons of the κ^1^-pytz ligand gives rise to a singlet resonance at δ 5.06. 2D NOESY analysis shows that this resonance displays NOE connections with a new doublet at δ 6.86 and a singlet resonance at δ 7.56 corresponding to the benzylic phenyl *ortho*-H and triazole ring protons for the κ^1^-pytz ligand of **2**, respectively. NMR assignment of signals corresponding to the formation of **2** are corroborated by sampling of the NMR solution for electrospray mass spectrometry analysis which reveals ions corresponding to the cation [Ru(pytz)_3_(NCCD_3_)]^2+^ (*m*/*z* 427.1356) and the ion-pair {[Ru(pytz)_3_(NCCD_3_)](PF_6_)}^+^ (*m*/*z* 999.2353) (Figure S10).

**Figure 5:**
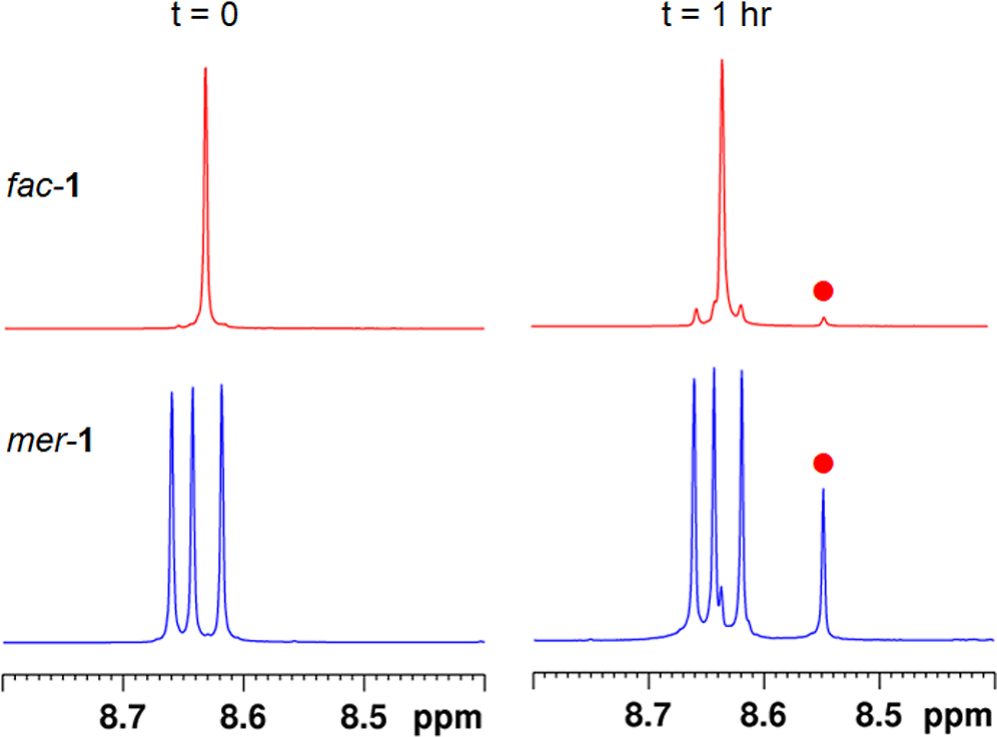
^1^H NMR spectra of *fac*-**1** and *mer*-**1** in *d*_3_-acetonitrile before and after 1 h of photolysis (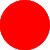 = **2**—*trans*-[Ru(κ^2^-pytz)_2_(κ^1^-pytz)(NCCD_3_)]^2+^).

In contrast, the ^1^H NMR spectrum of a sample of *fac*-**1** after 20 min is largely unchanged and lacks any obvious new resonances, in particular resonances for **2**, in agreement with our reactivity predictions. Close examination of the spectrum at 20 min irradiation, however, reveals the appearance of small singlet resonances at δ 8.66 and 8.62 that straddle the triazole proton resonance for *fac*-**1** and which correspond to the positions of triazole ring proton resonances of *mer*-**1**. Thus, as was an expected possibility for *fac*-**1**, the complex does indeed undergo slow photoisomerisation to yield *mer*-**1**. After 1 h of irradiation the resonances for *mer*-**1** become more prominent and small signals are now also discernible for the presence of **2**, presumably formed from *mer*-**1** after its formation via photoisomerisation of *fac*-**1** (Figure 5).

After an hour of irradiation of *mer*-**1**, signals for **2** have significantly grown in intensity while a singlet resonance at δ 8.63 is now clearly discernible (Figure 5) corresponding to the triazole ring protons of *fac*-**1** demonstrating the *fac*/*mer* isomerization process to be reversible. Between 4 and 8 h of irradiation both samples exhibit resonances for **2** as well as significant proportions of both isomers of **1**.

Interestingly, in addition to the κ^2^-pytz resonance for **2** at δ 9.88, a further very weak doublet resonance corresponding to a pytz pyridine H6 proton is observed at δ 10.35. The ratio of intensities of these two resonances appears to remain constant at approximately 1:0.02. It should be noted that formation of **2** could occur with coordination of the κ^1^-pytz ligand by either the triazole (**2**) or pyridine donor (**2**_Py_). We assign the dominant species as being the linkage isomer of **2** in which the monodentate pytz is bound by the triazole ring and tentatively assign the minor species as the pyridine-bound isomer. DFT calculations of the ground states for the two possible isomers show that the triazole bound isomer is more stable by 0.24 eV (23 kJ mol^–1^). This appears to be primarily due to the pendant pyridine ring imparting less steric strain and distortion in the approximately planar Ru(κ^2^-pytz)_2_ fragment upon coordination by the 5-membered heterocycle than is the case for the pendant triazole ring upon coordination by the pyridine ring (Figure S11).

**Figure 6:**
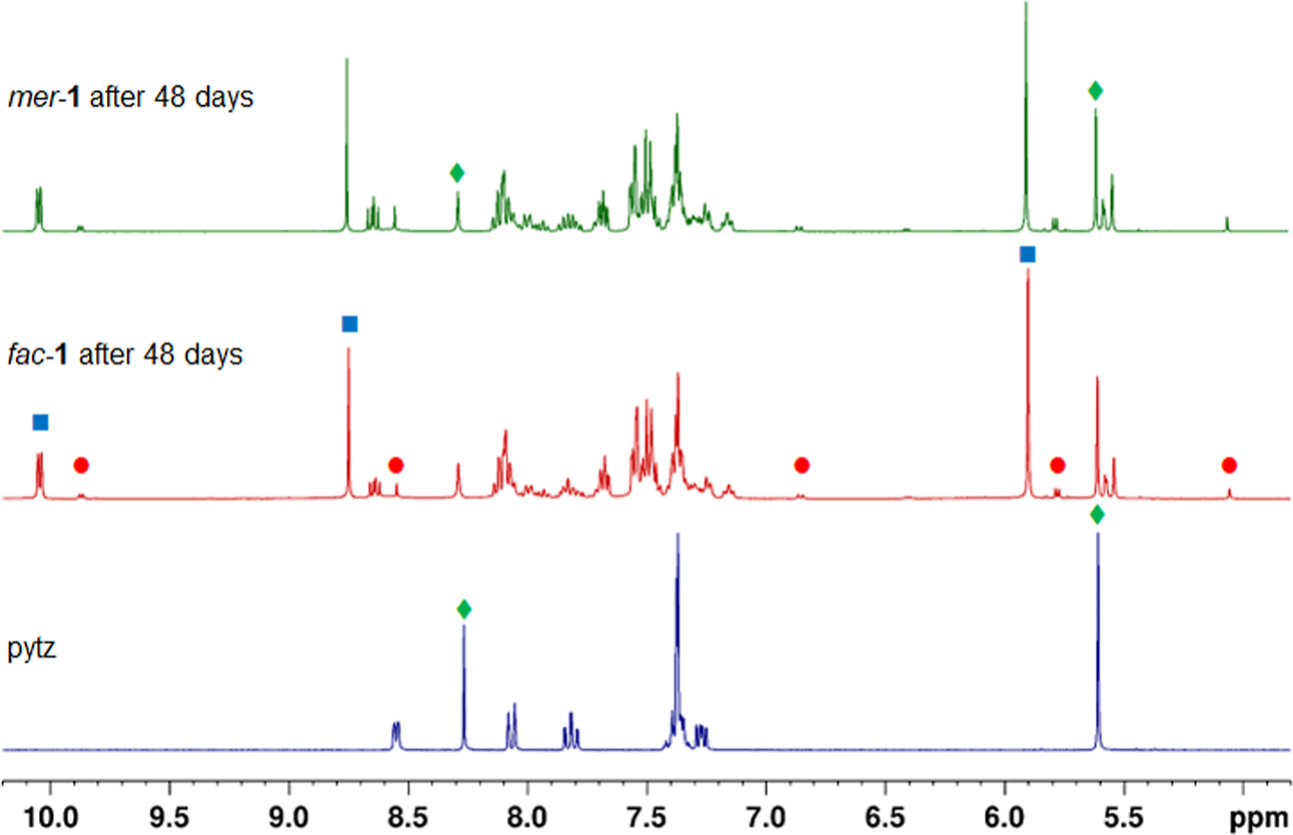
^1^H NMR spectra after prolonged photolysis of *fac*-**1** and *mer*-**1** in d_3_-acetonitrile along with reference spectrum of the ligand pytz in the same solvent (● = **2**, ■ = **3**, ⧫= pytz).

After 24 h of photolysis spectra for the two samples show a near identical composition with resonances present for both *fac*- & *mer*-**1** and **2** as well as those for another new species. These include a new pyridyl resonance at δ 10.05 (doublet, *J* = 5.3 Hz, py-H6) and pytz triazole and benzylic methylene singlet resonances at δ 8.75 and 5.91 respectively. Based on the singlet resonance for the methylene group this species is assigned as the *C*_2*h*_ symmetrical *bis*-solvent ligand-loss product *trans*-[Ru(κ^2^-pytz)_2_(NCMe)_2_]^2+^ (**3**) (Scheme 2). Interestingly, signals for free pytz are not immediately obvious as those for **3** begin to grow in, however, closer examination of the ^1^H NMR spectra at this point do reveal the appearance of a rather broad feature at 8.3 ppm coincident with the position of the triazole ring proton resonance for a reference sample of the free ligand (Figure 6). Upon continued irradiation, this signal as well as those for **3** continue to grow in intensity with those for free pytz remaining broadened, possibly due to limited solubility. Samples originating from *mer*-**1** and *fac*-**1** were left to undergo photolysis with periodic monitoring for a period of 48 days (Figure 6); while **3** becomes the dominant species, resonances are still clearly observed for *fac*/*mer*-**1** and **2** as well as free pytz in an approximate ratio of 1:0.08:0.08:0.24 of **3**:**2**:*fac*-**1**:*mer*-**1**. During this prolonged period of photolysis, very little change in the composition of the mixture was observed indicating that both pytz dechelation and ligand-loss may be photochemically reversible under these conditions and that a photochemical equilibrium is established (though thermally activated reversal cannot be entirely ruled out). Mass spectrometry analysis of the solutions at this point enables detection of ions corresponding to **1**, **2** and **3** (Figure S10).

**Figure 7:**
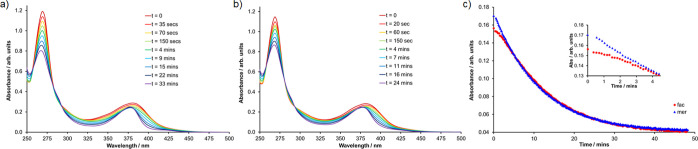
UV–visible absorption spectra recorded during the photolysis (λ^irr^ = 446 nm) of *fac*-**1** (a) and *mer*-**1** (b) and kinetic profiles for the absorbance intensity at 400 nm for both complexes (c, inset: kinetic profile over the first 4 min of photolysis).

The photochemical reactivities of both *fac*-**1** and *mer*-**1** were also monitored by UV–visible absorption spectroscopy with irradiation from a blue LED (446 nm, Figure S12). Under the much more dilute conditions required for optical spectroscopy, photolysis proceeds more rapidly due to the lack of inner filter effects that are present at the far higher concentrations required for NMR spectroscopy. UV–visible absorption spectra recorded during irradiation are shown in Figure 7. In both cases, the ^1^MLCT band is observed to reduce in intensity, narrow and undergo a slight blue-shift in the position of its maximum to ∼ 380 nm. Nonisosbestic behavior is observed, consistent with the divergent and multistep photochemistry identified by NMR spectroscopy. Kinetic traces for the absorbance intensity at 400 nm shows that over the first 4 min of photolysis, *fac*-**1** shows a much slower rate of reaction compared to *mer*-**1** (Figure 7c). Thereafter, the samples derived from the two isomers appear to show near-identical kinetic behavior. This is interpreted as being due to the fact that while *fac*-**1** appears to be unreactive to dechelation, it nevertheless undergoes photoisomerization to *mer*-**1**. Thus, for *fac*-**1** an initial induction period is observed during which *fac*-**1** and *mer*-**1** are photoequilibrated with the latter then able to undergo photodechelation to form **2**. Spectra were observed to cease evolution after approximately half an hour of irradiation. Due to the divergent and multistep nature of the photochemistry and the near identical behavior of the two samples after the initial short induction period, photochemical quantum yields derived from the data were anticipated to be unreliable indicators of the relative reactivity of the two isomers of **1** and so were not determined.

**Figure 8:**
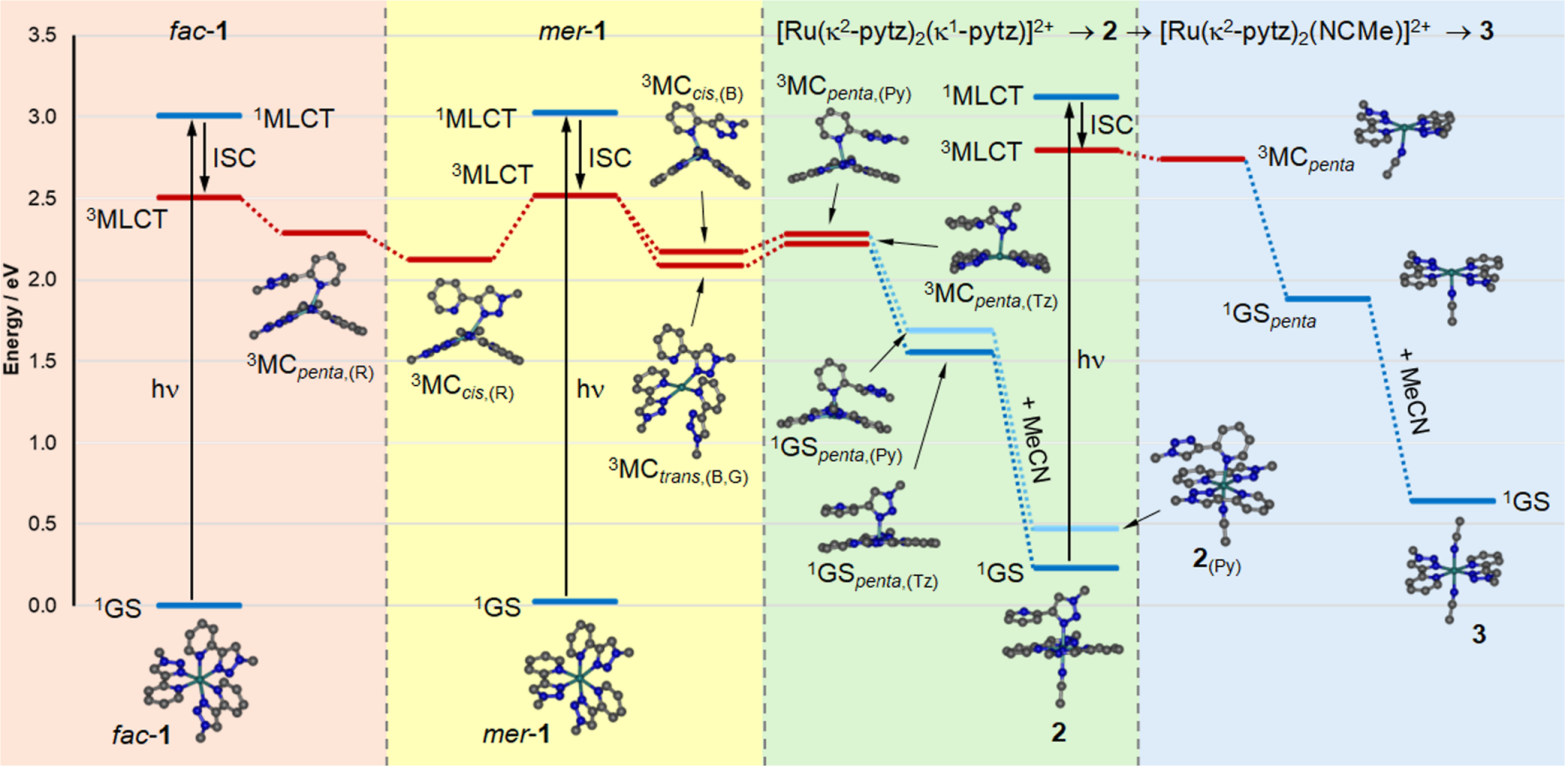
Potential photochemical reaction mechanism based on DFT calculations for the photoisomerization of *fac*-**1** and *mer*-**1** and the sequential photochemical formation of **2** and **3** from *mer*-**1**. Energy levels in blue represent singlet states while those in red represent triplet states. All energy levels represent optimized geometries with the exception of the ^1^MLCT states which are derived from TDDFT vertical excitation energies for S_1_ states at the relevant ground state geometries. All energies are quoted relative to that of *fac*-**1**.

To gain deeper insight into the photochemical reactivity of *fac*- & *mer*-**1** we carried out further DFT calculations to locate minima for ^3^MC states for the two isomers. Our previous work has highlighted the expansive nature of the ^3^MC state region of the lowest triplet state potential energy surfaces for [Ru(N^N)_3_]^2+^ complexes.^29,31,32^ This fairly shallow potential energy surface is often characterized by multiple ^3^MC state minima separated by relatively low energy barriers. While various possible routes through this potential energy landscape are feasible, a potential mechanistic picture for the *fac*/*mer* isomerization of **1** and the photodechelation of *mer*-**1** to form **2** is depicted in Figure 8. Using appropriate initial starting geometries, based on structural parameters derived from hexacoordinate ^3^MC_trans_ and ^3^MC_cis_ states, and pentacoordinate ^3^MC_penta_ states investigated in previous work, we were able to optimize the geometries of multiple ^3^MC states. Structural parameters and ruthenium atom spin densities are reported in Table 2 along with those for the ground and ^3^MLCT states already discussed. All of these ^3^MC states were found to lie significantly lower in energy than the ^3^MLCT states of the two isomers, accounting for the lack of luminescence at room temperature as well as at 77 K (vide supra).

As alluded to above, while the three *trans* N–Ru–N axes for *fac*-**1** are equivalent thus allowing for one unique ^3^MC_trans_ state, *mer*-**1** exhibits three inequivalent N–Ru–N axes and thus will allow for three distinct ^3^MC_trans_ states. Indeed, all four of these states for the two isomers were located and adopt elongation of two mutually *trans* Ru–N bonds to lengths of 2.45 to 2.54 Å (Table 2). Ruthenium atom spin densities are between 1.89 and 1.91 indicating the presence of two unpaired electrons at the metal center. Analysis of the SONOs shows that in each case the lower energy SONO has ruthenium d-orbital character while the higher energy SONO has d_*z*^2^_-like dσ* antibonding character (Figure S13).

Minima were also sought for further ^3^MC states where again the three unique ligands in *mer*-**1** present the possibility of three distinct ^3^MC_cis_ states compared to the one possible state for *fac*-**1**. For *mer*-**1**, two ^3^MC_cis_ states characterized by elongation of the two Ru–N bonds to the pytz ligands colored blue and red in Table 2 were located (^3^MC_cis,(B)_ and ^3^MC_cis,(R)_) while attempted optimization of the remaining state ^3^MC_cis,(G)_ was unsuccessful leading to relaxation as a ^3^MC_trans_ state. ^3^MC_cis,(B)_ and ^3^MC_cis,(R)_ states of *mer*-**1** are characterized by ruthenium atom spin densities of 1.79 and 1.77 respectively and have higher energy SONOs of d_x_^2^_–y_^2^-like dσ* character (Figure S14). Ru–N bond lengths for the pytz(B) and pytz(R) ligands of 2.23 to 2.63 Å are observed (Table 2). For *fac*-**1**, a ^3^MC_cis_ state could not be optimized, however, a related pentacoordinate ^3^MC state (^3^MC_penta,(R)_) was found with one monodentate pytz ligand bound by the pyridine donor (Ru–N = 2.21 Å) and a pendant triazole moiety (Table 2 and Figure S15).

Concerning the photoisomerization of *fac*- and *mer*-**1**, we note that a rotation of the pytz(R) ligand of the *mer* isomer by approximately 180° will result in formation of the *fac* isomer. The ^3^MC_cis,(R)_ state of *mer*-**1** would therefore be heavily implicated for its involvement in this process. It is possible that population of ^3^MC_cis,(R)_ from the ^3^MLCT state following photoexcitation and resultant repulsion of pytz(R) could allow for evolution of the geometry to yield ^3^MC_penta,(R)_ from which reverse intersystem crossing and rechelation would result in formation of *fac*-**1** (Figure 8). The reverse sequence via depopulation of the *fac*-**1**^3^MLCT state would account for the *fac* → *mer* isomerization process. Similar photoisomerization routes and ^3^MC_penta_ states have been proposed for *fac*/*mer* isomerization in cyclometalated iridium(III) complexes.^42^

Regarding onward photochemistry of *mer*-**1** toward formation of **2**, ^3^MC_cis,(R)_ would not be expected to contribute as colpanarisation of the two bidentate pytz ligands (blue and green) would be sterically hindered. However, ^3^MC_cis,(B)_ could facilitate such a coplanarisation of the red and green pytz ligands. The Ru–N bond to the pyridine donor of pytz (blue) (2.23 Å) is markedly shorter than the corresponding bond for the triazole donor (2.63 Å) and could evolve to form a pentacoordinate pyridine bound ^3^MC_penta,(Py)_ state (Figures 8 and S15) from which intersystem crossing would lead to a ground state pentacoordinate species (^1^GS_penta(Py)_, Figures 8 and S16), which could capture a solvent ligand and form **2**_(Py)_. While ^3^MC_cis,(G)_ could not be located, a pentacoordinate triazole-bound ^3^MC state was found (^3^MC_penta,(Tz)_) which could result from evolution from a ^3^MC_trans_ state, such as ^3^MC_trans,(B,G)_ (as depicted in Figure 8) or ^3^MC_trans,(R,B)_. Subsequent intersystem crossing to the singlet manifold and coordination of a solvent ligand would then yield **2** which as noted earlier is 0.24 eV more stable than **2**_(Py)_ and likely the dominant intermediate species observed experimentally.

Similarly to *fac*- and *mer*-**1**, the HOMO and LUMO of **2** are dominated by metal d-orbital and κ^2^-pytz π* character respectively (Figure S17). The formal dissociation of pytz from **2** in forming **3** is also a photochemically mediated process. TDDFT calculations (Figure S18 and Table S2) reveal that the Franck–Condon S_1_ state of **2** lies 2.89 eV above its ground state and has ^1^MLCT state character. Optimisation of the ^3^MLCT state of **2** (Figure S19) was carried out and this lies 2.67 eV higher in energy than the optimized ground state of **2**. Dissociation of the monodentate pytz ligand in the triplet state would result in formation of a pentacoordinate species of the form [Ru(pytz)_2_(NCMe)]^2+^ which has ^3^MC_penta_ state character (Figures 8 and S20). Intersystem crossing to the singlet state followed by coordination of a second solvent ligand will then form **3**. Overall, the formation of **3** is calculated to be endothermic by 0.62 eV relative to *mer*-**1**.

It should be noted that photolysis of **2** could lead to either loss of pytz or the solvent ligand. The ^3^MC_penta,(Tz)_ for [Ru(κ^2^-pytz)_2_(κ^1^-pytz)]^2+^ resulting from loss of acetonitrile from **2** lies 0.49 eV below the ^3^MC_penta_ state for [Ru(pytz)_2_(NCMe)]^2+^ resulting from loss of the κ^1^-pytz ligand (Figure 8). This could account for the relative slowness of photolysis of **2** to form **3** where photochemical exchange of the solvent ligand may dominate over photorelease of pytz. This could also allow for a route to reform *mer*-**1** from **2** as would seemingly occur during NMR spectroscopic monitoring of photolysis. Further, photochemical solvent ligand loss from **3** could under the high concentrations required for NMR spectroscopy allow for recoordination of free pytz to reform **2**. Taken together, this could account for the convergence to a static reaction mixture composition that was observed during prolonged photolysis.

## Conclusions

The efficiency and stereochemical outcomes of photochemical reactions of metal complexes are affected by many factors including the stereochemistry of the initial complex, the solvent and also coordinating/noncoordinating counterions. However, prior knowledge of the geometrical character of available and possible ^3^MC states, their preferential roles in mediating ground state recovery or promoting photochemical reactivity, and the intramolecular steric interactions which will favor or disfavor the population of particular ^3^MC states can provide useful insights into likely outcomes of photoexcitation and onward photochemistry. Here, we have shown that the facial and meridional isomers of the homoleptic complex [Ru(pytz)_3_]^2+^ exhibit very different photochemical reactivities due to favorability of ^3^MC_cis_ state-mediated spectator ligand coplanarisation during pytz dechelation for the *mer*-isomer which is inhibited for the *fac*-isomer. The findings complement those regarding the fundamental differences in photophysical properties of isomers of luminescent complexes relevant to device applications.^45−47^ Here, the data provide important insights on the impact that seemingly subtle differences in the structure of complexes can have on photochemical reactivity and are of relevance in areas including the design of new photoreactive systems, for example, for application in photoactivated chemotherapeutics.

## Experimental

### General Methods

Unless otherwise stated, all reagents were purchased from commercial sources and used without further purification. pytz^48^ and [Ru(η^6^-cymene)(pytz)(Cl)][PF_6_]^49^ were prepared as previously described. ^1^H and ^13^C NMR spectra were recorded on Bruker 400 and 600 Avance NMR spectrometers at 298 K. Chemical shifts (δ) are reported in parts per million (ppm) and referenced to residual solvent peaks (CD_3_CN: ^1^H δ 1.94 ppm, ^13^C δ 118.26, 1.32 ppm). Coupling constants (*J*) are reported in Hertz (Hz). Electrospray mass spectra (HRMS-ESI) were collected on an Agilent 1290 Infinity II instrument with 6545 QTOF. UV–visible absorption spectra were recorded on an Agilent Cary-60 spectrophotometer utilizing quartz cuvettes of 10 mm path length.

### Electrochemistry

Cyclic voltammograms were measured using a PalmSens EmStat3 potentiostat with PSTrace electrochemical software. Analyte solutions with a typical concentration of 1.5 mmol dm^–3^ were prepared using dry MeCN, freshly distilled from CaH_2_. The supporting electrolyte was NBu_4_PF_6_, being recrystallized from EtOH and oven-dried prior to use with a typical solution concentration of 0.2 mol dm^–3^. The working electrode was a glassy carbon disc, Pt wire was used as a counter electrode and the reference electrode was Ag/AgCl, being chemically isolated from the analyte solution by an electrolyte-containing bridge tube tipped with a porous frit. All potentials are quoted relative to the Fc^+^/Fc couple as an internal reference.

### Photochemistry

Photolysis experiments were carried out by irradiating the appropriate solutions contained within either NMR tubes or 10 mm path length quartz cuvettes with a blue LED (17 mW, 446 nm) or compact 23 W fluorescent light bulb (Hg). Samples were maintained at room temperature (25 °C) throughout the measurements with the aid of a Peltier temperature-controlled cuvette holder or an electronic fan (NMR samples).

### Synthesis of [Ru(pytz)_3_][PF_6_]_2_

[Ru(η^6^-cymene)(pytz)(Cl)][PF_6_] (155 mg, 0.238 mmol), pytz (119 mg, 0.504 mmol) and NaPF_6_ (58 mg, 0.345 mmol) were combined in deaerated 3:1 (v/v) EtOH/H_2_O (40 mL) and heated to 90 °C in the dark under an N_2_ atmosphere for 36 h. Upon cooling, an aqueous solution of NH_4_PF_6_ (78 mg, 0.478 mmol) (15 mL) was added to ensure complete precipitation of the bright yellow product which was subsequently collected by filtration, washed with Et_2_O and dried in vacuo. No further purification was required. ^1^H NMR analysis reveals the product to be composed of a mixture of *mer*- and *fac*-isomers in a 1:0.8 respective ratio. Yield = 245 mg, 94%. ^1^H NMR (*d*_3_-MeCN, 400 MHz, δ): 5.54 (s), 5.56–5.59 (m), 7.12–7.20 (m), 7.21–7.28 (m), 7.28–7.42 (m), 7.71 (d), 7.76–7.83 (m), 7.90–8.04 (m), 8.04–8.11 (m), 8.61 (s), 8.63 (s), 8.64 (s), 8.65 (s). HRMS (ESI) calcd for RuC_42_H_36_N_12_PF_6_ [M–PF_6_]^+^: *m*/*z* = 955.1866, found *m*/*z* = 955.1901; calcd for RuC_42_H_36_N_12_ [M]^2+^: *m*/*z* = 405.1109, found *m*/*z* = 405.1131.

### Separation of *mer*- and *fac*-Isomers

*mer*- and *fac*-Isomers of **1** could not be satisfactorily separated by column chromatography owing to streaking and persistent coelution of the two bright yellow-colored fractions. However, separation of small quantities of *mer*- and *fac*-**1** was achieved via preparative thin layer chromatography. Typically, a 40–50 mg portion of *rac*-**1** was dissolved in MeCN and loaded onto a 20 × 20 cm, 1500 μM thickness, SiO_2_ preparative TLC plate. Elution was performed first with CH_2_Cl_2_, and then after drying, second with a 5:1 (v/v) CH_2_Cl_2_/acetone solvent system. Two closely spaced bright yellow-colored bands were observed, the first upper band being the *mer*-isomer, the second lower band corresponding to the *fac*-isomer. The bands were scraped from the plate (taking care to avoid any regions of potential overlap), with *mer*- and *fac*-**1** being desorbed from the SiO_2_ by sonication and dissolution into MeCN. The solutions were then filtered to remove spent silica and evaporated to dryness. This procedure typically afforded ca. 15 mg of each isomer.

### *mer*-[Ru(pytz)_3_][PF_6_]_2_

^1^H NMR (*d*_3_-MeCN, 400 MHz, δ): 5.54 (s, 2H), 5.56–5.59 (m, 4H), 7.14–7.19 (m, 2H), 7.21–7.27 (m, 6H), 7.30–7.42 (m, 10H), 7.70 (d, *J* = 5.5 Hz, 2H), 7.77 (d, *J* = 5.6 Hz, 1H), 7.90–8.04 (m, 5H), 8.08 (d, *J* = 7.8 Hz, 1H), 8.62 (s, 1H), 8.64 (s, 1H), 8.66 (s, 1H). ^13^C NMR (*d*_3_-MeCN, 151 MHz): δ 56.25, 56.50, 56.54, 122.81, 122.97, 123.63, 125.75, 126.03, 126.07, 126.32, 126.60, 126.83, 128.78, 129.25, 129.30, 129.87, 130.01, 130.04, 130.07, 130.09, 134.58, 134.63, 135.00, 138.90, 138.95, 139.07, 149.06, 149.34, 149.37, 152.12, 152.28, 152.40, 153.22, 153.54.

### *fac*-[Ru(pytz)_3_][PF_6_]_2_

^1^H NMR (*d*_3_-MeCN, 400 MHz): δ 5.54 (s, 6H), 7.12–7.17 (m, 6H), 7.27–7.41 (m, 12H), 7.80 (d, *J* = 5.5 Hz, 3H), 7.99 (t, *J* = 7.5 Hz, 3H), 8.06 (d, *J* = 8.0 Hz, 3H), 8.63 (s, 3H). ^13^C NMR (*d*_3_-MeCN, 151 MHz, δ): 56.30, 123.36, 125.99, 126.71, 128.79, 129.87, 130.04, 135.04, 139.15, 149.16, 152.26, 153.16.

### Computational Details

The geometries of the ground states of complexes **1** to **3** were optimized using DFT using the B3LYP hybrid functional^50,51^ as implemented in the Orca 4.2.1 software package.^52,53^ Def2-ECP effective core potential and def2/j auxiliary basis set were used for ruthenium with def2-tzvp(-f) basis sets used for all other atoms.^54^ All calculations were conducted using Grimme’s D3-BJ dispersion correction^55,56^ along with the SMD implicit solvation model (acetonitrile).^57^ In these DFT calculations the resolution-of-identity (RI) approximation for hybrid functionals (as implemented in ORCA) was employed to calculate the Coulomb energy term using the Ahlrichs/Weigend Def2-TZV basis as the auxiliary basis set and the exchange term by the so-called ‘chain-of-spheres exchange’ (COSX) algorithm. The benzyl substituents of the triazole rings were replaced by methyl groups as these will have little impact on the photophysical properties and also saves on computational expense. The ^3^MLCT states of the complexes were optimized by unrestricted DFT starting from the ground state geometries whereas ^3^MC_trans_ and ^3^MC_cis_ states were optimized from initial guess geometries whose key bond lengths and angles were informed by previous data on related complexes.^30,31^ Molecular orbitals were visualized using the Gabedit software package with isosurfaces set to 0.02.

## Supporting Information Available

^1^H and ^13^C NMR spectra, mass spectrometry data, cyclic voltammograms and computational data for complexes **1**, **2**, **3** (PDF)
Cartesian coordinates of all calculated minima (ZIP)
