# Supplementary material for: Prediction and Rationalization of Different Photochemical Behaviors of mer- and fac-Isomers of [Ru(pyridyltriazole)3]2+
Source: Inorg Chem. 2024 Sep 5;63(37):17287–97. doi: 10.1021/acs.inorgchem.4c03154 (PMC11409217; doi:10.1021/acs.inorgchem.4c03154)
Supplement: Supplementary file 1 — ic4c03154_si_001.pdf [file ic4c03154_si_001.pdf]

Supporting Information for

Prediction and rationalisation of different photochemical behaviours of *mer*- and *fac*-isomers of $[Ru(pyridyltriazole)_3]^{2+}$

Paul A. Scattergood* and Paul I.P. Elliott*

Department of Chemistry and Centre for Functional Materials, University of Huddersfield, Queensgate, Huddersfield, HD1 3DH, UK

E-mail: p.scattergood@hud.ac.uk; p.i.elliott@hud.ac.uk

Contents

**Figure S1**	^{1}H NMR spectrum of *mer*-$[Ru(PyTz)_3][PF_6]_2$ (*mer*-**1**)	**S2**
**Figure S2**	^{13}C NMR spectrum of *mer*-$[Ru(PyTz)_3][PF_6]_2$ (*mer*-**1**)	**S2**
**Figure S3**	^{1}H NMR spectrum of *fac*-$[Ru(PyTz)_3][PF_6]_2$ (*fac*-**1**)	**S3**
**Figure S4**	^{13}C NMR spectrum of *fac*-$[Ru(PyTz)_3][PF_6]_2$.(*fac*-**1**)	**S3**
**Figure S5**	Cyclic voltammograms recorded for *mer*- and *fac*-**1**	**S4**
**Figure S6**	Selected computed molecular orbital plots for *mer*- and *fac*-**1**	**S4**
**Figure S7**	TDDFT-calculated electronic absorbance spectra for *mer*- and *fac*-**1**	**S5**
**Table S1**	Selected TDDFT-calculated transitions for *fac*-**1** and *mer*-**1**	**S6**
**Figure S8**	SONO plots for the T_1 ^{3}MLCT states of *fac*-**1** and *mer*-1	**S9**
**Figure S9**	Spectral output profile of 23 W CFL irradiation source	**S9**
**Figure S10**	Electrospray mass spectrometry data recorded during the photolysis of **1**	**S10**
**Figure S11**	Optimised geometries of **2** and $\mathbf{2}_{(Py)}$	**S10**
**Figure S12**	Spectral output profile of blue LED irradiation source	**S10**
**Figure S13**	SONO plots for the $^3MC_{trans}$ states of *fac*-**1** and *mer*-**1**	**S11**
**Figure S14**	SONO plots for the $^3MC_{cis}$ states of *mer*-**1**	**S11**
**Figure S15**	SONO plots for the $^3MC_{penta}$ states of *fac*-**1** and *mer*-**1**	**S12**
**Figure S16**	Molecular orbital plots for the $^1GS_{penta}$ species $[Ru(\kappa^2\text{-pytz})_2(\kappa^1\text{-pytz})]^{2+}$(**2**)	**S12**
**Figure S17**	Selected calculated molecular orbital plots for **2**	**S13**
**Figure S18**	TDDFT-calculated electronic absorbance spectra for **2**	**S13**
**Table S2**	Selected TDDFT-calculated transitions for **2**	**S14**
**Figure S19**	SONO plots for the ^{3}MLCT state **2**	**S14**
**Figure S20**	SONO plots for the $^3MC_{penta}$ state for the species $[Ru(\kappa^2\text{-pytz})_2(NCMe)]^{2+}$	**S15**
**Figure S21**	Molecular orbital plots for the GS species $[Ru(\kappa^2 pytz)_2(NCMe)]^{2+}$.	**S15**
**Figure S22**	Selected calculated molecular orbital plots for **3**	**S15**

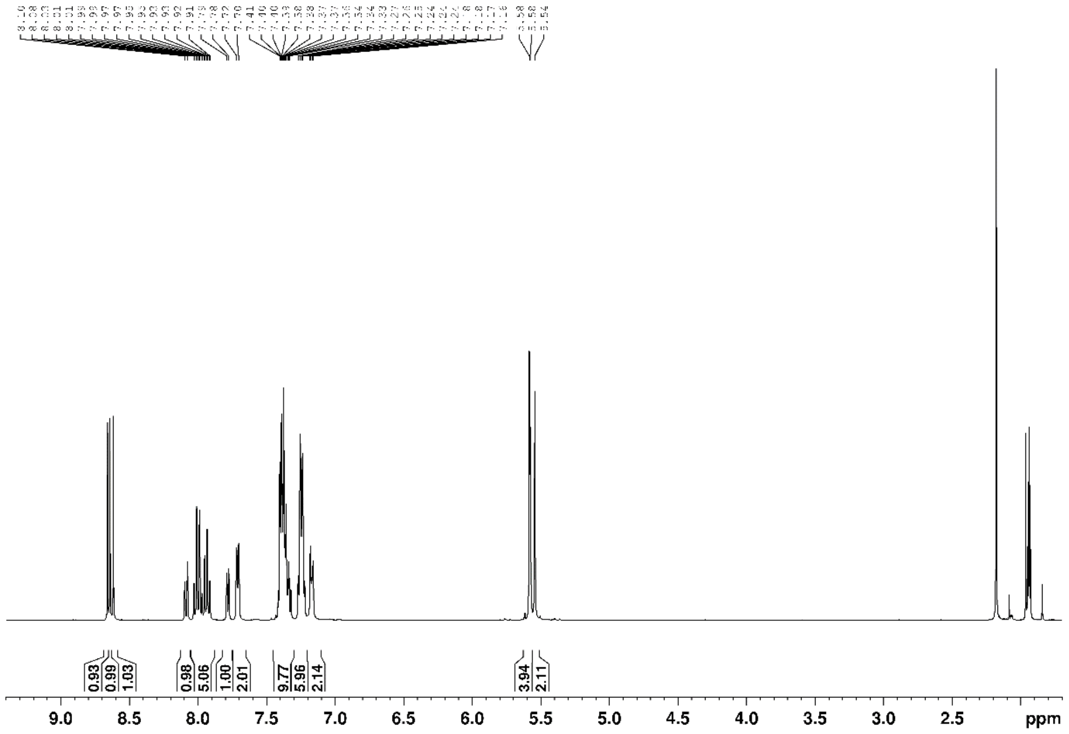

Figure S1 ^{1}H NMR (d_3-MeCN, 400 MHz) spectrum of *mer*-[Ru(PyTz)$_3$][PF$_6$]$_2$

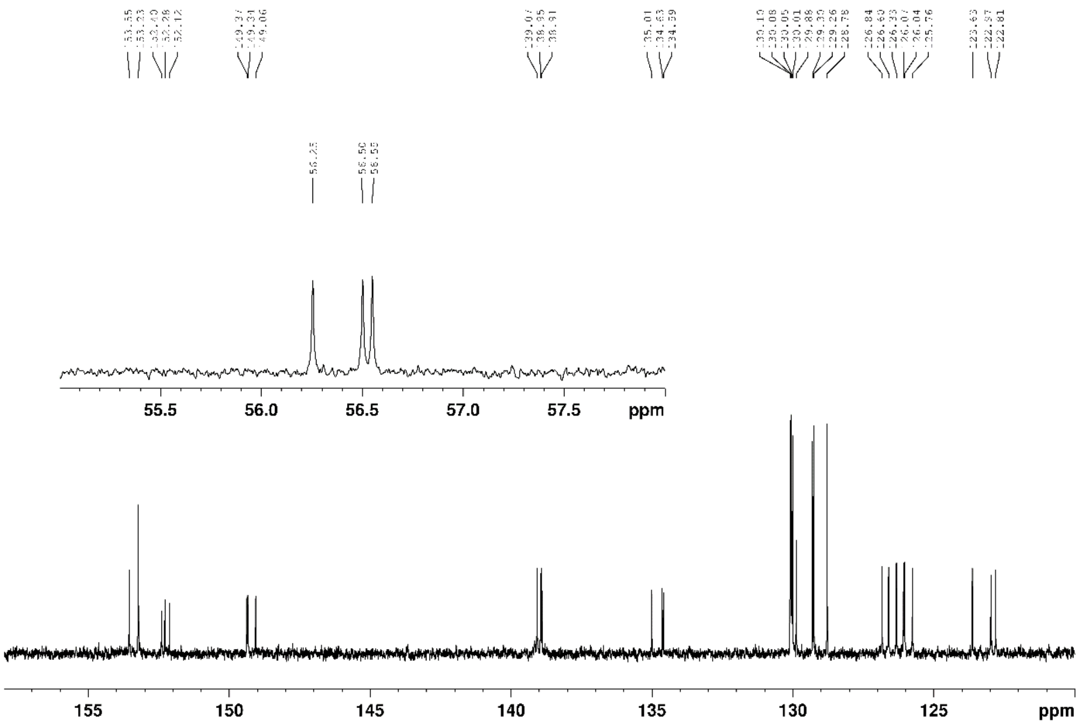

Figure S2 ^{13}C NMR (d_3-MeCN, 151 MHz) spectrum of *mer*-[Ru(PyTz)$_3$][PF$_6$]$_2$

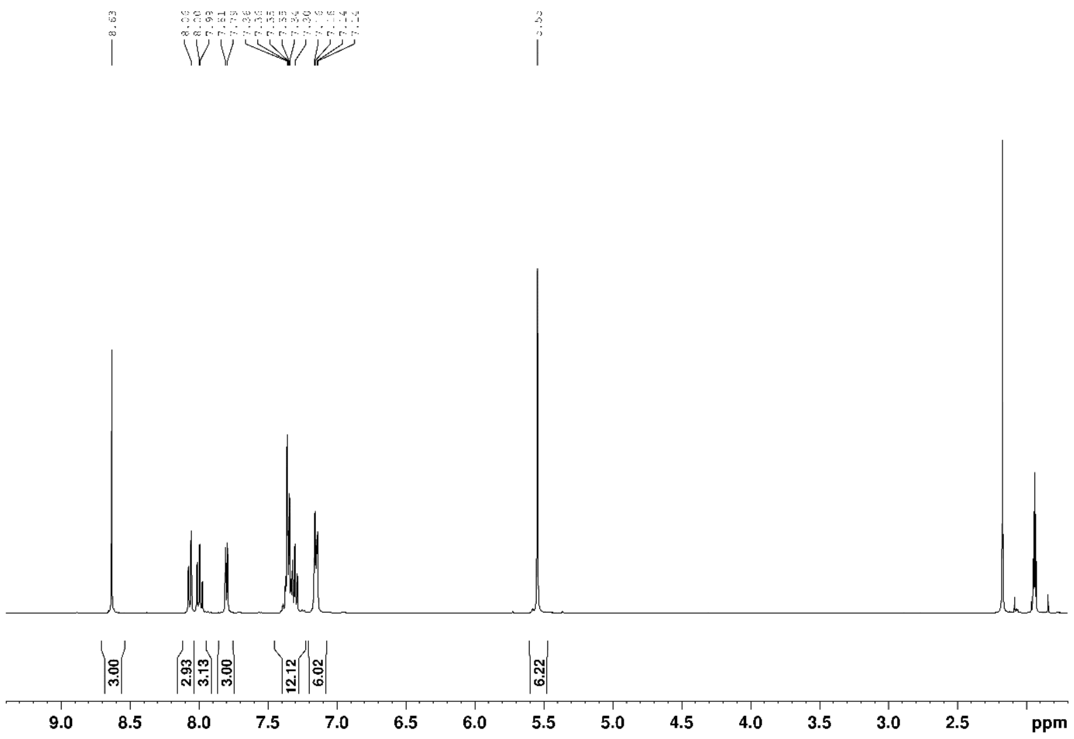

Figure S3 ^{1}H NMR (d_3-MeCN, 400 MHz) spectrum of *fac*-[Ru(PyTz)$_3$][PF$_6$]$_2$

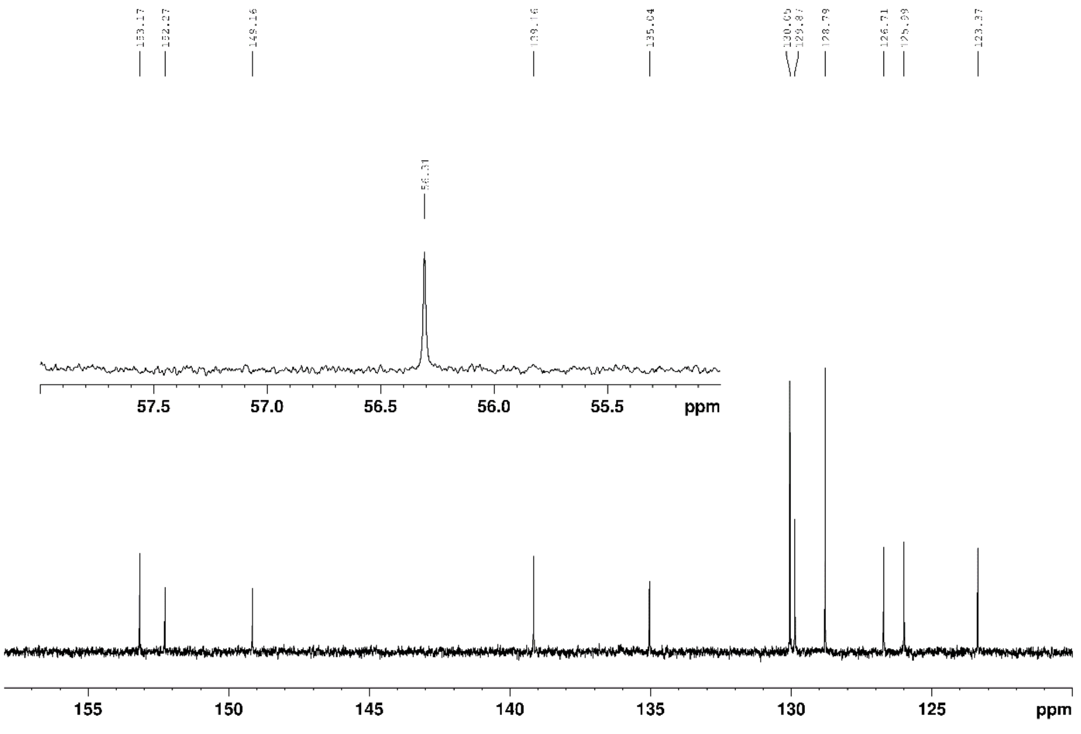

Figure S4 ^{13}C NMR (d_3-MeCN, 151 MHz) spectrum of *fac*-[Ru(PyTz)$_3$][PF$_6$]$_2$

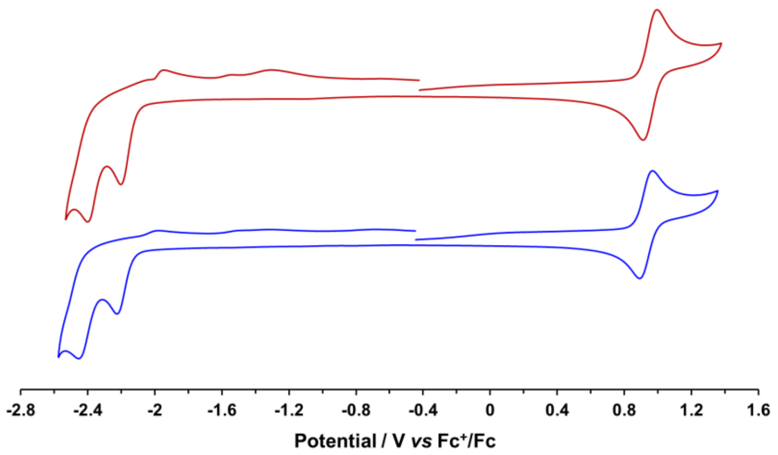

Figure S5. Cyclic voltammetry traces for *mer*-**1** (blue) and *fac*-**1** (red) recorded at 100 mVs^{-1} in acetonitrile solution with $^{n}Bu_4NPF_6$ as supporting electrolyte.

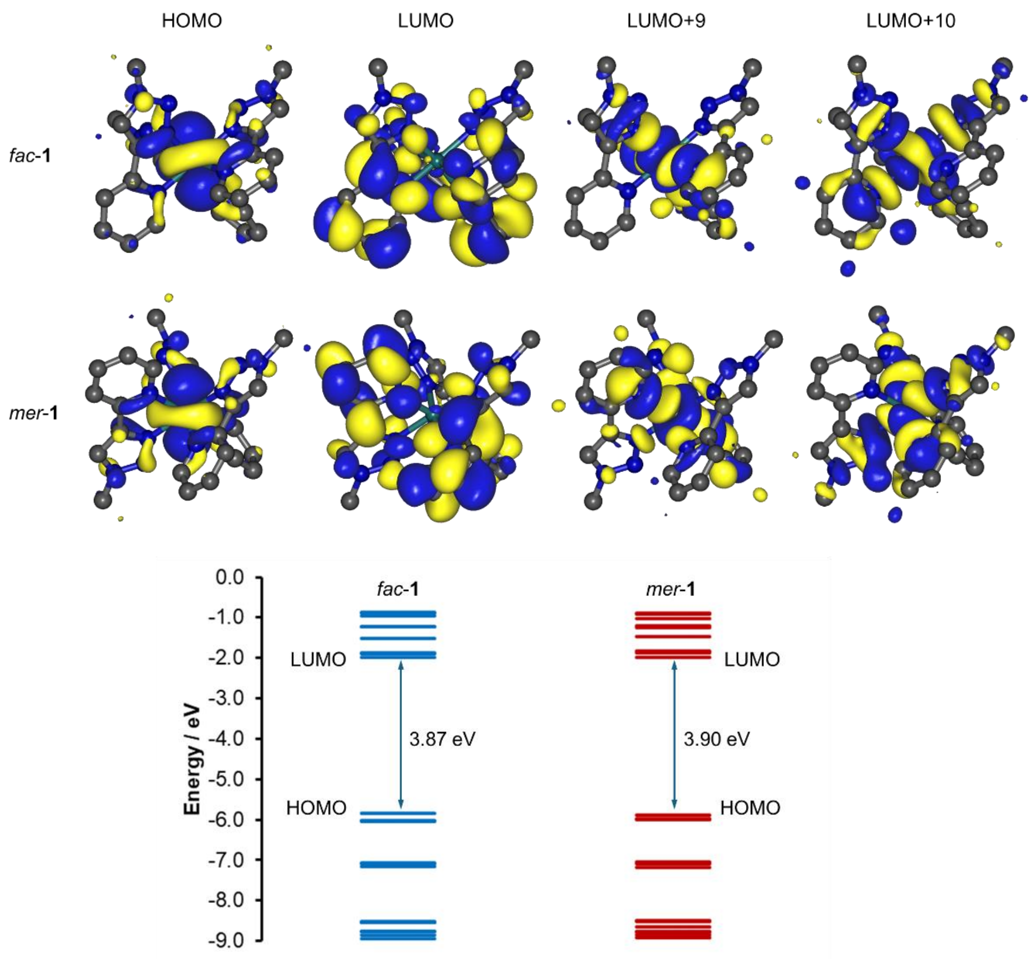

Figure S6. Top: isosurface plots (0.02 a.u.) of the HOMO and LUMO for *fac*- and *mer*-**1** along with plots of their dσ* orbitals (LUMO+9 and +10 in both cases). Bottom: molecular orbital energy level diagram for *fac*- and *mer*-**1**.

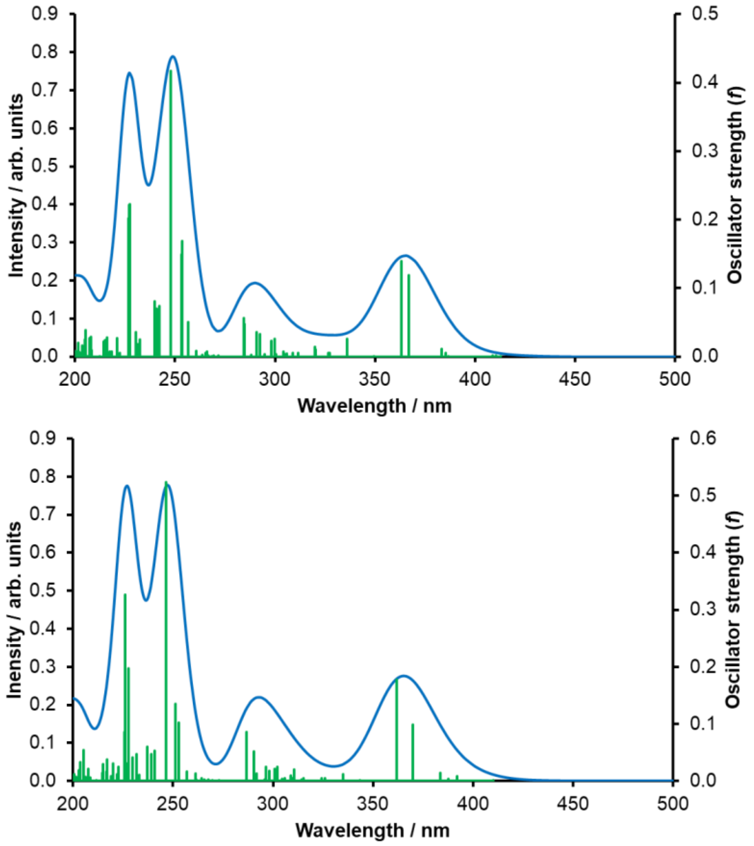

Figure S7. Calculated UV-visible absorption spectra for *fac*-**1** (top) and *mer*-**1** (bottom) from TDDFT calculations. The blue traces used 0.15 eV full-width at half-maximum line broadening.

Table S1. Selected TDDFT transitions for *fac*-**1** and *mer*-**1** and their dominant composition and character.

State	Energy / cm^{-1}	λ / nm	*f*(osc)	Composition	Character
			fac-**1**		
S_1	24230	413	0.000913	HOMO→LUMO (64 %)<br>HOMO→LUMO+1 (33 %)	^{1}MLCT(py)
S_7	27268	367	0.119	HOMO-1→LUMO (37 %)<br>HOMO-2→LUMO+1 (20 %)	^{1}MLCT(py)
S_8	27537	363	0.140	HOMO-2→LUMO+2 (25 %)<br>HOMO-2→LUMO+1 (19 %)<br>HOMO-1→LUMO+1 (18 %)<br>HOMO-2→LUMO (17 %)	^{1}MLCT(py)
S_{10}	29754	336	0.0257	HOMO→LUMO+3 (36 %)<br>HOMO-1→LUMO+2 (23 %)<br>HOMO-2→LUMO+1 (19 %)	^{1}MLCT(py)
S_{22}	33359	300	0.0264	HOMO→LUMO+7 (72 %)	^{1}MLCT(tz)
S_{23}	33536	298	0.0231	HOMO→LUMO+8 (67 %)	^{1}MLCT(tz)
S_{25}	34185	293	0.0329	HOMO-1→LUMO+6 (62 %)	^{1}MLCT(tz)
S_{26}	34409	291	0.0357	HOMO-2→LUMO+6 (56 %)	^{1}MLCT(tz)
S_{28}	35116	285	0.048	HOMO-2→LUMO+8 (20 %)<br>HOMO-1→LUMO+8 (17 %)<br>HOMO-2→LUMO+7 (16 %)	^{1}MLCT(tz)
S_{29}	35184	284	0.0563	HOMO-1→LUMO+7 (19 %)<br>HOMO-1→LUMO+8 (18 %)<br>HOMO-2→LUMO+7 (17 %)<br>HOMO-2→LUMO+8 (16 %)	^{1}MLCT(tz)
S_{39}	38994	256	0.0508	HOMO-2→LUMO+10 (32 %)<br>HOMO-1→LUMO+9 (21 %)	^{1}MC
S_{40}	39449	254	0.169	HOMO-3→LUMO+3 (17 %)<br>HOMO-5→LUMO+1 (17 %)<br>HOMO-3→LUMO+1 (16 %)	^{1}LC
S_{41}	39501	253	0.149	HOMO-5→LUMO+2 (34 %)<br>HOMO-4→LUMO+3 (19 %)	^{1}LC
S_{42}	40379	248	0.417	HOMO-4→LUMO+2 (14 %)<br>HOMO-5→LUMO (14 %)<br>HOMO-5→LUMO+3 (10 %)<br>HOMO-3→LUMO+1 (10 %)	^{1}LC
S_{43}	41290	242	0.0741	HOMO-3→LUMO+3 (67 %)	^{1}LC
S_{44}	41541	241	0.0701	HOMO-4→LUMO+3 (60 %)	^{1}LC
S_{45}	41705	240	0.0805	HOMO-5→LUMO+3 (65 %)	^{1}LC
S_{46}	43055	232	0.0245	HOMO-3→LUMO+4 (62 %)<br>HOMO-5→LUMO+5 (16 %)	^{1}LC
S_{47}	43227	231	0.018	HOMO-4→LUMO+5 (43 %)<br>HOMO-4→LUMO+4 (22 %)<br>HOMO-5→LUMO+4 (19 %)	^{1}LC
S_{48}	43395	230	0.0357	HOMO-5→LUMO+5 (42 %)<br>HOMO-4→LUMO+4 (17 %)<br>HOMO-3→LUMO+5 (14 %)	^{1}LC
S_{49}	43996	227	0.222	HOMO-3→LUMO+5 (41 %)<br>HOMO-5→LUMO+5 (21 %)	^{1}LC
S_{50}	44059	227	0.221	HOMO-5→LUMO+4 (43 %)<br>HOMO-4→LUMO+4 (23 %)	^{1}LC

State	Energy / cm^{-1}	λ / nm	*f*(osc)	Composition	Character
S_{51}	44095	227	0.202	HOMO-5→LUMO+5 (24 %)	^{1}LC
				HOMO-5→LUMO+4 (18 %)	
				HOMO-4→LUMO+5 (15 %)	
T_1	21583	463		HOMO→LUMO+1 (87 %)	^{3}MLCT(py)
T_2	20657	484		HOMO→LUMO+2 (74 %)	^{3}MLCT(py)
T_3	22175	451		HOMO→LUMO (58 %)	^{3}MLCT(py)
T_4	23292	429		HOMO-1→LUMO+2 (40 %)	^{3}MLCT(py)
				HOMO-2→LUMO+1 (27 %)	
				HOMO-1→LUMO (24 %)	
T_5	23556	425		HOMO-1→LUMO+1 (36 %)	^{3}MLCT(py)
				HOMO-2→LUMO+2 (27 %)	
				mer-**1**	
S_1	24384	410	0.000885	HOMO→LUMO (93 %)	^{1}MLCT(py)
S_7	27030	370	0.099	HOMO-1→LUMO+1 (30 %)	^{1}MLCT(py)
				HOMO-2→LUMO+2 (23 %)	
				HOMO-2→LUMO (22 %)	
				HOMO-1→LUMO (17 %)	
S_8	27643	362	0.178	HOMO-1→LUMO+2 (43 %)	^{1}MLCT(py)
				HOMO-2→LUMO+1 (41 %)	
S_{16}	32190	311	0.0203	HOMO-1→LUMO+4 (71 %)	^{1}MLCT(py)
S_{21}	33115	302	0.0251	HOMO→LUMO+6 (36 %)	^{1}MLCT(tz)
				HOMO-2→LUMO+6 (18 %)	
S_{22}	33247	301	0.0211	HOMO→LUMO+7 (32 %)	^{1}MLCT(tz)/^{1}MC
				HOMO→LUMO+10 (15 %)	
				HOMO-2→LUMO+10 (10 %)	
S_{23}	33525	298	0.018	HOMO-1→LUMO+6 (35 %)	^{1}MLCT(tz)
				HOMO→LUMO+8 (18 %)	
S_{24}	33721	297	0.025	HOMO→LUMO+8 (38 %)	^{1}MLCT(tz)
				HOMO→LUMO+7 (19 %)	
				HOMO-2→LUMO+8 (18 %)	
S_{26}	34291	292	0.0136	HOMO-2→LUMO+7 (58 %)	^{1}MLCT(tz)
S_{28}	34442	290	0.0518	HOMO-2→LUMO+6 (35 %)	^{1}MLCT(tz)
				HOMO-1→LUMO+8 (17 %)	
				HOMO-1→LUMO+6 (13 %)	
S_{29}	34897	287	0.0854	HOMO-1→LUMO+7 (30 %)	^{1}MLCT(tz)
				HOMO-2→LUMO+8 (22 %)	
				HOMO→LUMO+8 (12 %)	
S_{39}	38940	257	0.0163	HOMO-1→LUMO+10 (30 %)	^{1}MC/^{1}LC
				HOMO-5→LUMO+2 (12 %)	
S_{40}	39539	253	0.103	HOMO-5→LUMO+1 (27 %)	^{1}LC
				HOMO-4→LUMO+3 (15 %)	
				HOMO-5→LUMO+2 (12 %)	
S_{41}	39830	251	0.135	HOMO-5→LUMO+2 (22 %)	^{1}LC
				HOMO-3→LUMO+3 (12 %)	
				HOMO-4→LUMO+2 (11 %)	
S_{42}	40576	247	0.523	HOMO-5→LUMO (18 %)	^{1}LC
				HOMO-4→LUMO+1 (14 %)	
				HOMO-4→LUMO+2 (12 %)	
S_{43}	41528	241	0.0525	HOMO-3→LUMO+3 (71 %)	^{1}LC
S_{44}	41842	239	0.0474	HOMO-4→LUMO+3 (56 %)	^{1}LC
				HOMO-5→LUMO+3 (13 %)	

State	Energy / cm^{-1}	λ / nm	*f*(osc)	Composition	Character
S_{45}	42147	237	0.0604	HOMO-5→LUMO+3 (58 %)<br>HOMO-4→LUMO+5 (13 %)<br>HOMO-4→LUMO+3 (12 %)	^{1}LC
S_{47}	43167	232	0.0466	HOMO-3→LUMO+4 (44 %)<br>HOMO-4→LUMO+5 (25 %)<br>HOMO-5→LUMO+4 (15 %)	^{1}LC
S_{48}	43552	230	0.041	HOMO-4→LUMO+5 (28 %)<br>HOMO-3→LUMO+5 (26 %)<br>HOMO-5→LUMO+5 (15 %)	^{1}LC
S_{49}	43943	228	0.197	HOMO-3→LUMO+6 (24 %)<br>HOMO-3→LUMO+5 (19 %)<br>HOMO-4→LUMO+5 (15 %)	^{1}LC
S_{50}	44077	227	0.0308	HOMO-5→LUMO+4 (33 %)<br>HOMO-3→LUMO+6 (29 %)	^{1}LC
S_{51}	44232	226	0.326	HOMO-4→LUMO+5 (20 %)<br>HOMO-5→LUMO+5 (17 %)<br>HOMO-3→LUMO+6 (16 %)	^{1}LC
T_1	20600	485		HOMO→LUMO+1 (25 %)<br>HOMO-1→LUMO+1 (16 %)<br>HOMO→LUMO (15 %)	^{3}MLCT(py)
T_2	21409	467		HOMO→LUMO (49 %)<br>HOMO→LUMO+2 (13 %)	^{3}MLCT(py)
T_3	22634	442		HOMO→LUMO+1 (52 %)<br>HOMO-1→LUMO+1 (28 %)	^{3}MLCT(py)
T_4	22892	437		HOMO→LUMO+2 (47 %)<br>HOMO-1→LUMO (24 %)<br>HOMO→LUMO (11 %)	^{3}MLCT)py)
T_5	21567	464		HOMO-2→LUMO (27 %)<br>HOMO-2→LUMO+2 (21 %)<br>HOMO-1→LUMO (16 %)<br>HOMO→LUMO+2 (14 %)	^{3}MLCT(py)

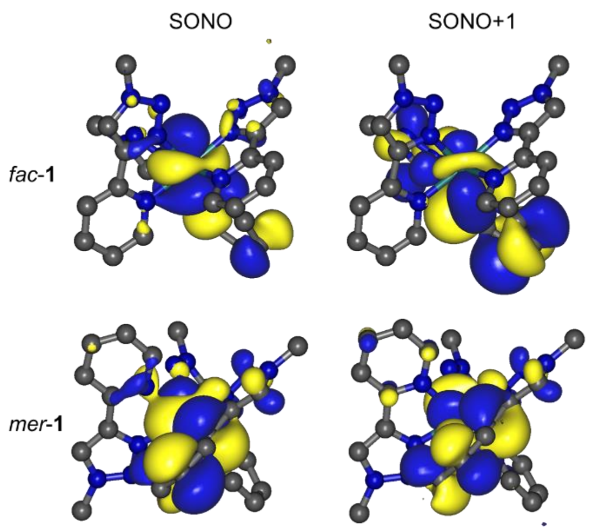

Figure S8. Isosurface plots (0.02 a.u.) of the lower lying and higher lying singly occupied natural orbitals (SONO and SONO+1 respectively) for the T_1 ^{3}MLCT states of *fac*-**1** and *mer*-**1**.

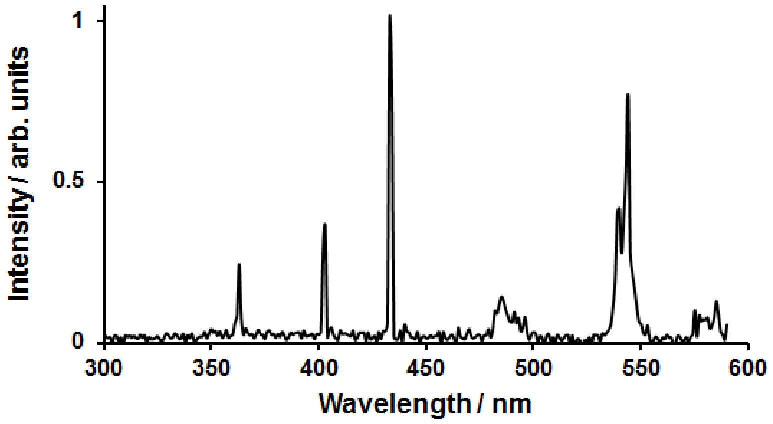

Figure S9. Spectral output profile of the 23W Compact Fluorescent Lamp irradiation source employed to study photochemical reactivity monitored by ^{1}H NMR spectroscopy.

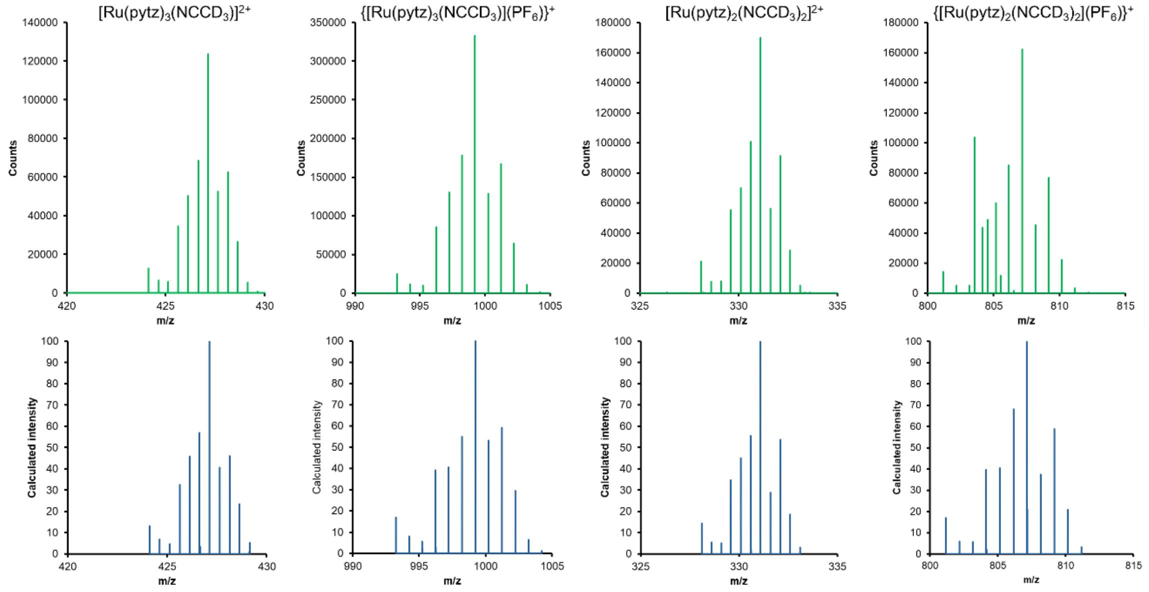

Figure S10. Electrospray mass spectra showing ions for **2** ($[Ru(pytz)_3(NCCD_3)]^{2+}$ & $\{[Ru(pytz)_3(NCCD_3)](PF_6)\}^+$ after 1 hour photolysis) and **3** ($[Ru(pytz)_2(NCCD_3)_2]^{2+}$ & $\{[Ru(pytz)_2(NCCD_3)_2](PF_6)\}^+$ after 1 week photolysis) in d_3-acetonitrile solutions during photolysis of **1**. Experimental spectra in green, calculated spectra in blue).

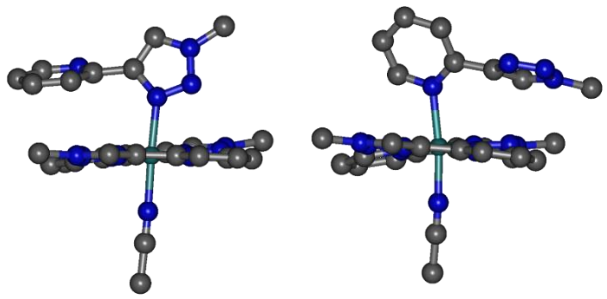

2 $\mathbf{2}_{(Py)}$

Figure S11. Geometries of **2** and $\mathbf{2}_{(Py)}$ showing the greater structural distortion due to steric strain in the Ru(κ^2-pytz)$_2$ fragment in $\mathbf{2}_{(Py)}$ compared to **2**.

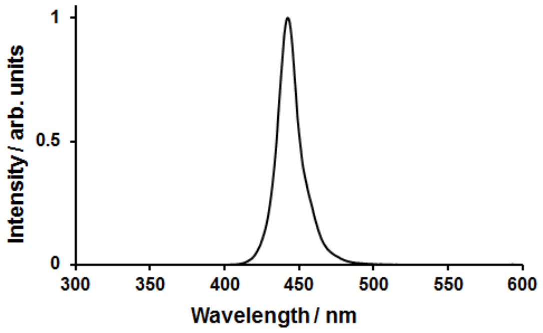

Figure S12. Spectral output profile of the blue LED irradiation source employed to study photochemical reactivity monitored by electronic absorption spectroscopy.

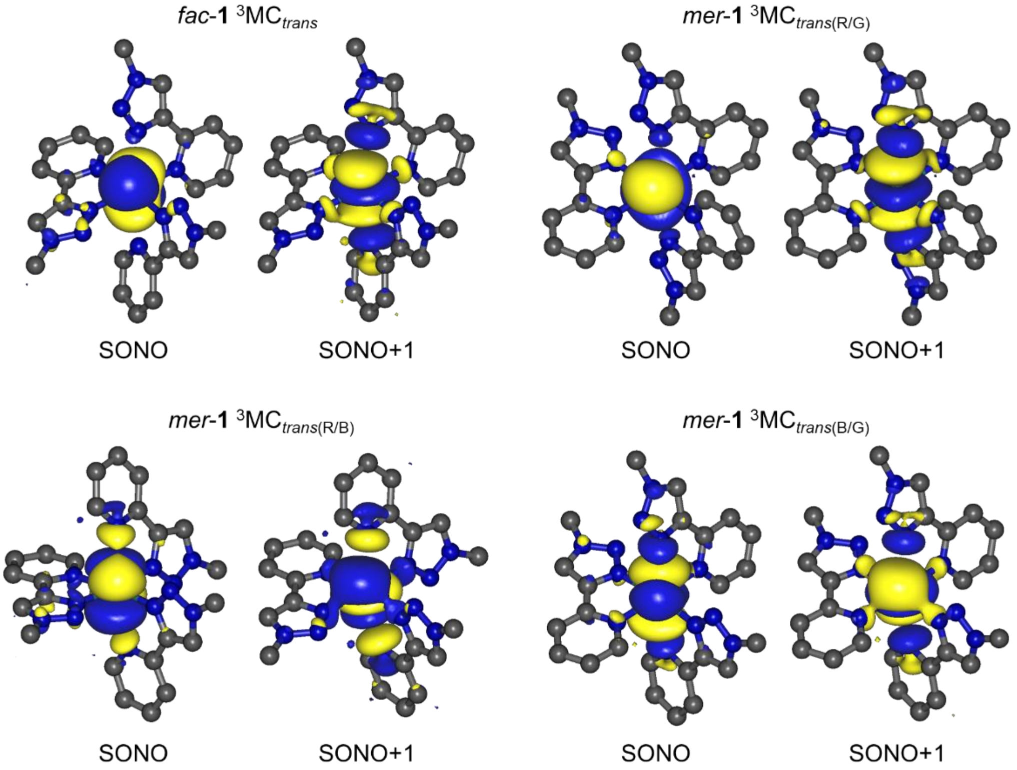

Figure S13. Isosurface plots (0.02 a.u.) of the lower lying and higher lying singly occupied natural orbitals (SONO and SONO+1 respectively) for the $^3MC_{trans}$ states of *fac*-**1** and *mer*-**1**.

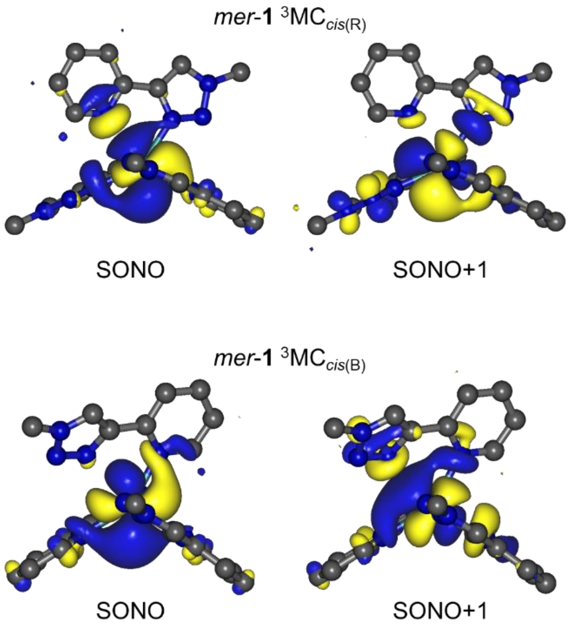

Figure S14. Isosurface plots (0.02 a.u.) of the lower lying and higher lying singly occupied natural orbitals (SONO and SONO+1 respectively) for the $^3MC_{cis}$ states of *mer*-**1**.

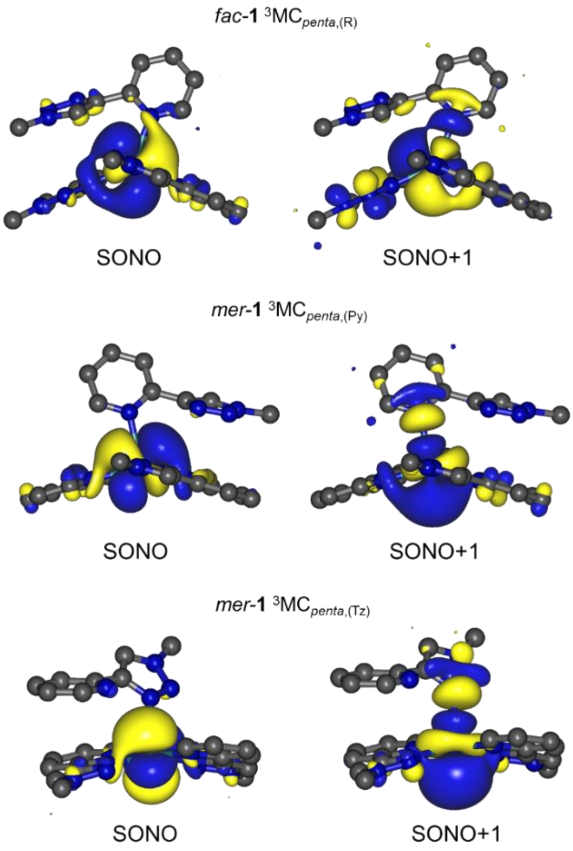

Figure S15. Isosurface plots (0.02 a.u.) of the lower lying and higher lying singly occupied natural orbitals (SONO and SONO+1 respectively) for the $^{3}MC_{penta}$ states of *fac*-**1** and *mer*-**1**.

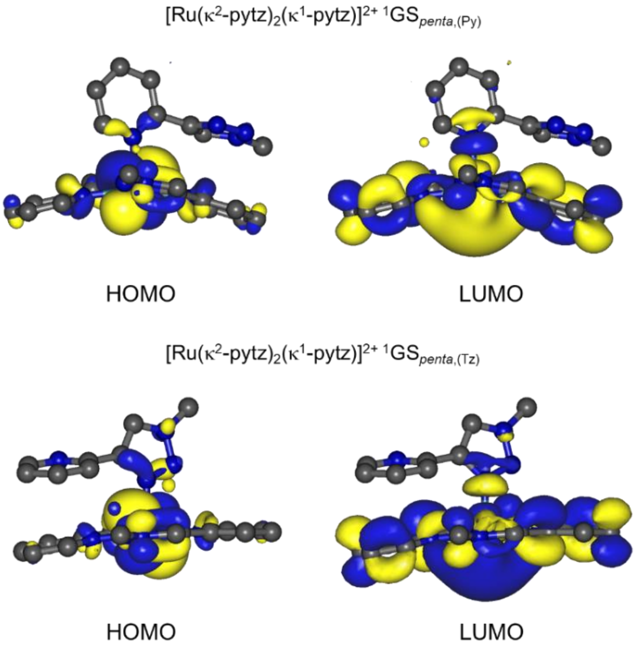

Figure S16. Isosurface plots (0.02 a.u.) of the HOMO and LUMO for the $^{1}GS_{penta}$ species $[Ru(\kappa^{2}\text{-pytz})_{2}(\kappa^{1}\text{-pytz})]^{2+}$ in which the monodentate ligand is either coordinated by pyridine (top) or triazole (bottom).

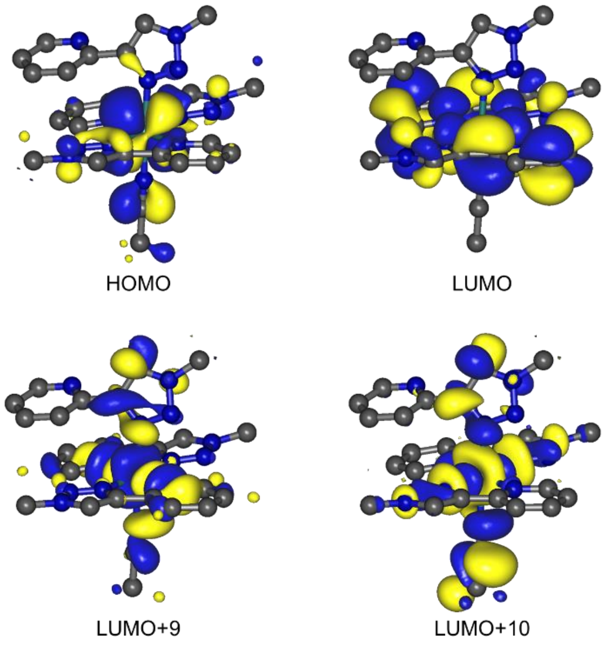

Figure S17. Isosurface plots (0.02 a.u.) of the HOMO and LUMO for **2** along with plots of the dσ* orbitals, LUMO+9 and +10.

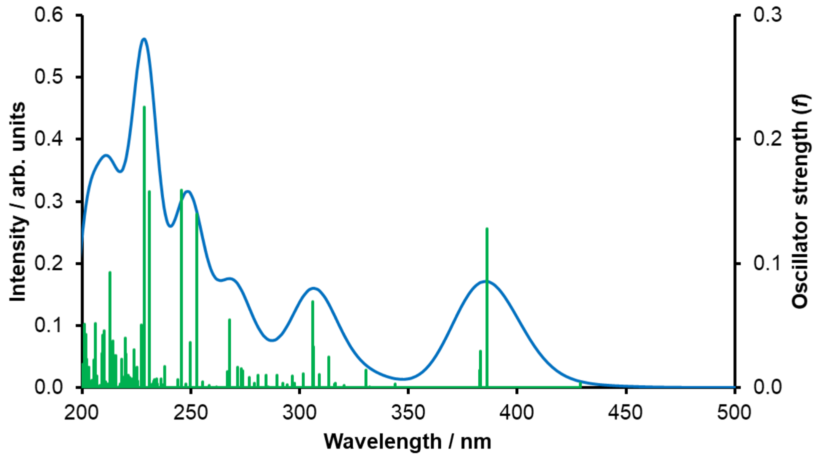

Figure S18. Calculated UV-visible absorption spectrum for **2** from TDDFT. The blue trace used 0.15 eV full-width at half-maximum line broadening.

Table S2. Selected TDDFT transitions for **2** and their dominant composition and character.

State	Energy / cm^{-1}	λ / nm	*f*(osc)	Composition	Character
S_1	23313	429	0.00339	HOMO→LUMO (99 %)	^{1}MLCT(κ^2-pytz)
S_2	25906	386	0.128	HOMO-1→LUMO (76 %)<br>HOMO-2→LUMO (20 %)	^{1}MLCT(κ^2-pytz)
S_3	26110	383	0.0296	HOMO-2→LUMO (75 %)<br>HOMO-1→LUMO (15 %)	^{1}MLCT(κ^2-pytz)
S_{12}	31909	313	0.0245	HOMO-1→LUMO+3 (33 %)<br>HOMO→LUMO+10 (14 %)<br>HOMO-1→LUMO+9 (10 %)	^{1}MLCT(κ^1-pytz)/^{1}MC
S_{14}	32651	306	0.0327	HOMO→LUMO+5 (29 %)<br>HOMO-2→LUMO+9 (24 %)<br>HOMO-2→LUMO+10 (15 %)	^{1}MLCT(κ^2&κ^1-pytz)/^{1}MC
S_{15}	32685	306	0.0694	HOMO→LUMO+5 (53 %)<br>HOMO-2→LUMO+10 (13 %)<br>HOMO-2→LUMO+9 (11 %)	^{1}MLCT(κ^2&κ^1-pytz)/^{1}MC
S_{31}	37340	268	0.055	HOMO-4→LUMO (56 %)<br>HOMO-3→LUMO+1 (18 %)	^{1}LC(κ^2-pytz)
S_{32}	37358	268	0.0453	HOMO-1→LUMO+9 (24 %)<br>HOMO-1→LUMO+7 (14 %)<br>HOMO→LUMO+9 (13 %)	^{1}MLCT(κ^1-pytz)/^{1}MC
S_{38}	39602	253	0.14	HOMO-3→LUMO+1 (40 %)<br>HOMO-5→LUMO+1 (21 %)	^{1}LC(κ^2-pytz)
S_{39}	40059	250	0.0363	HOMO-4→LUMO+1 (57 %)<br>HOMO-5→LUMO+1 (14 %)	^{1}LC(κ^2-pytz)
S_{41}	40731	246	0.159	HOMO-5→LUMO+1 (48 %)<br>HOMO-3→LUMO+1 (12 %)	^{1}LC(κ^2-pytz)
S_{53}	43340	231	0.158	HOMO-5→LUMO+3 (23 %)<br>HOMO-4→LUMO+2 (17 %)<br>HOMO-4→LUMO+4 (13 %)<br>HOMO-5→LUMO+2 (12 %)	^{1}LC(κ^2&κ^1-pytz)
S_{54}	43737	229	0.226	HOMO-3→LUMO+5 (22 %)<br>HOMO-3→LUMO+4 (18 %)<br>HOMO-5→LUMO+2 (14 %)	^{1}LC(κ^2&κ^1-pytz)

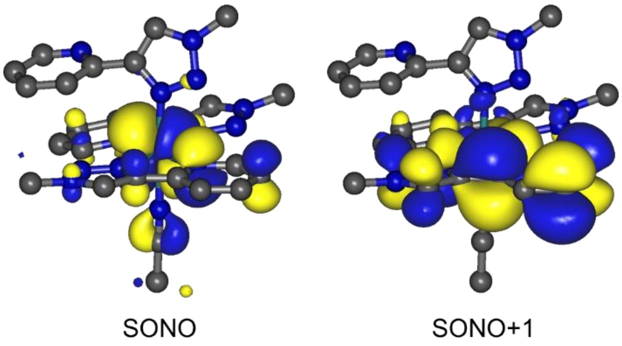

Figure S19. Isosurface plots (0.02 a.u.) of the lower lying and higher lying singly occupied natural orbitals (SONO and SONO+1 respectively) for the ^{3}MLCT state **2**.

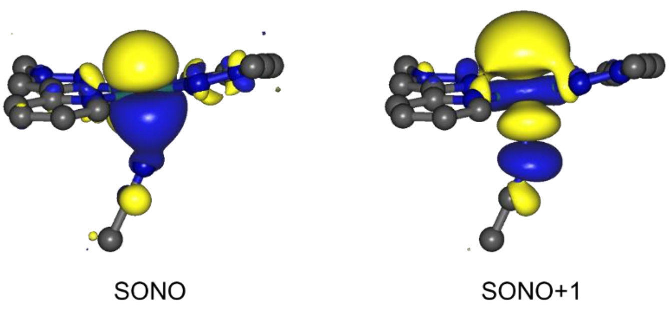

Figure S20. Isosurface plots (0.02 a.u.) of the lower lying and higher lying singly occupied natural orbitals (SONO and SONO+1 respectively) for the $^3MC_{penta}$ state for the species $[Ru(\kappa^2\text{-pytz})_2(NCMe)]^{2+}$.

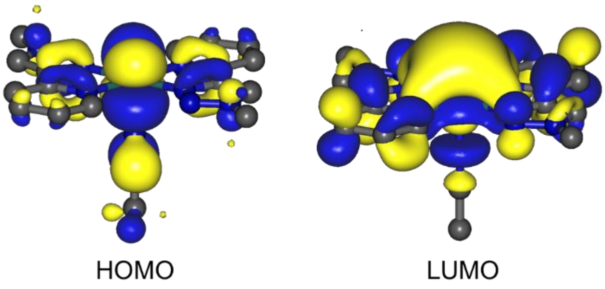

Figure S21. Isosurface plots (0.02 a.u.) of the HOMO and LUMO for the pentacoordinate ground state species $[Ru(\kappa^2\text{-pytz})_2(NCMe)]^{2+}$.

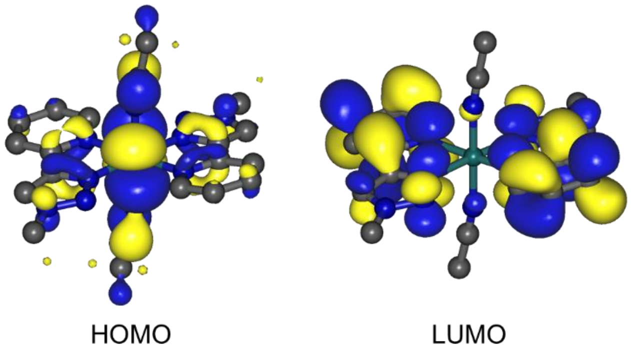

Figure S22. Isosurface plots (0.02 a.u.) of the HOMO and LUMO for **3**.
